# Multilineage hematopoietic recovery with concomitant antitumor effects using low dose Interleukin-12 in myelosuppressed tumor-bearing mice

**DOI:** 10.1186/1479-5876-6-26

**Published:** 2008-05-19

**Authors:** Lena A Basile, Timothy K Gallaher, Darryl Shibata, Joseph D Miller, Dan Douer

**Affiliations:** 1 Neumedicines Inc., 2275 East Foothill Blvd., Pasadena, California, USA; 2 University of Southern California, Keck School of Medicine, Health Sciences Campus, Los Angeles, California, USA

## Abstract

**Background:**

Interleukin-12 (IL-12) is a cytokine well known for its role in immunity. A lesser known function of IL-12 is its role in hematopoiesis. The promising data obtained in the preclinical models of antitumor immunotherapy raised hope that IL-12 could be a powerful therapeutic agent against cancer. However, excessive clinical toxicity, largely due to repeat dose regimens, and modest clinical response observed in the clinical trials have pointed to the necessity to design protocols that minimize toxicity without affecting the anti-tumor effect of IL-12. We have focused on the lesser known role of IL-12 in hematopoiesis and hypothesized that an important clinical role for IL-12 in cancer may be as an adjuvant hematological cancer therapy. In this putative clinical function, IL-12 is utilized for the prevention of cancer therapy-related cytopenias, while providing concomitant anti-tumor responses over and above responses observed with the primary therapy alone. This putative clinical function of IL-12 focuses on the dual role of IL-12 in hematopoiesis and immunity.

**Methods:**

We assessed the ability of IL-12 to facilitate hematopoietic recovery from radiation (625 rad) and chemotherapy (cyclophosphamide) in two tumor-bearing murine models, namely the EL4 lymphoma and the Lewis lung cancer models. Antitumor effects and changes in bone marrow cellularity were also assessed.

**Results:**

We show herein that carefully designed protocols, in mice, utilizing IL-12 as an adjuvant to radiation or chemotherapy yield facile and consistent, multilineage hematopoietic recovery from cancer therapy-induced cytopenias, as compared to vehicle and the clinically-utilized cytokine granulocyte colony-stimulating factor (G-CSF) (positive control), while still providing concomitant antitumor responses over and above the effects of the primary therapy alone. Moreover, our protocol design utilizes single, low doses of IL-12 that did not yield any apparent toxicity.

**Conclusion:**

Our results portend that despite its past failure, IL-12 appears to have significant clinical potential as a hematological adjuvant cancer therapy.

## Background

Radiation and chemotherapy generally exhibit a relatively narrow therapeutic index leading to significant toxicity to normal tissues even at the standard doses used in routine therapeutic regimens [1]. A common toxicity that limits the dose intensity of most cancer therapies is hematological toxicity.

A clear-cut correlation, however, between chemotherapeutic dose intensity and the probability of complete remission, relapse-free survival, and overall survival has been convincingly demonstrated for lymphomas, breast carcinoma and other chemosensitive tumors [2]. Remission and survival rates also are increased by dose-dense therapy. For example, dose-dense therapy followed by hematopoietic support in the form of transplanted hematopoietic progenitor and stem cells [3,4] has provided some success in treating various malignancies.

Additional support in the form of hematopoietic factors as an adjuvant therapy can be administered to counteract the hematological toxicity of currently practiced cancer regimens [5-10]. For example, radiation or chemotherapy often result in various treatment-induced cytopenias that can be counteracted by the administration of G-CSF to treat neutropenia [11] and EPO to treat anemia [12]. However, other countervailing treatments, targeted to the broad range of cytopenias resulting from the use of radiation or chemotherapy, are unavailable. Moreover, there are currently no available therapies, other than platelet transfusions, for the treatment of cancer-related thrombocytopenia [13,14]. Given the success in utilizing hematopoietic growth factors to address the hematological toxicity of various cancer therapies, it would appear that the discovery and development of novel hematological adjuvant therapies may yield more successful cancer treatment modalities, and in turn, may lead to increased cancer remission rates and overall patient survival.

Over the past several decades, numerous candidate hematopoietic growth factors or cytokines capable of multilineage hematopoietic recovery following myelosuppression in murine studies have come to light. These factors include Interleukin 1 (IL-1) [15-17], Stem Cell Factor (SCF or kit ligand) [18], Thrombopoietin (TPO) [19] and TPO derivatives, such as Megarkaryocyte Growth and Development Factor (Peg-rHuMGDF) [20]. In clinical trials, however, each of these factors failed for various reasons. For IL-1, relative clinical efficacy as compared to toxicity was the issue [10,21], and for SCF [22], TPO and TPO derivatives [23], the presence of serious adverse events in Phase I clinical trials halted further clinical development.

Interleukin-12 (IL-12) is well-known for its essential role in bridging the innate and adaptive arms of immunity, in regulating inflammatory responses, innate resistance to infection, and adaptive immunity [24]. Moreover, based on its potent antitumor responses in mice [25-29], IL-12 has been clinically evaluated as a single agent immunotherapy that possibly could provide clinically relevant antitumor responses in humans. IL-12 failed in clinical trials due to clinical toxicity, largely due to the repeat dose regimens utilized, and modest clinical responses [24,30]. Overall, results observed in the clinical trials have pointed to the necessity of designing protocols that minimize toxicity without affecting the antitumor effect of IL-12 [24].

Still another lesser known role of IL-12 is in the regulation of hematopoiesis [31-35], which appears to have been overshadowed by its well-studied role in immunity. We have sought to elucidate the lesser known hematological properties of IL-12. Overall, we have found that IL-12 possesses potent, hematopoietic recovery properties in the face of lethal and sublethal radiation [36]. In the present studies, we further demonstrate the potent and consistent hematological properties of IL-12 using carefully designed protocols that utilize radiation or chemotherapy (cyclophosphamide) in two myelosuppressed, tumor-bearing, murine model systems. As an internal, positive control, we also compare the IL-12-facilitated, multilineage hematopoietic recovery to that obtained with the clinically-utilized hematopoietic growth factor, granulocyte colony-stimulating factor (G-CSF). Importantly, the present studies demonstrate that the hematological properties of IL-12 are observed using single, low doses with no apparent toxicity. Concomitant with the hematological effect, we demonstrate that IL-12 exhibits antitumor effects that are over and above the effects of the primary therapy in the two tumor models studied. Interestingly, an unexpected and potent platelet recovery effect is also observed in these sublethal model systems, which further suggests a novel role for IL-12 in thrombopoiesis. Our findings portend that despite its past failure, IL-12 appears to have significant clinical potential as a hematological adjuvant cancer therapy.

## Methods

### Animals

Female and male C57BL/6 mice, age 4–5 weeks, were purchased from Harlan (Indianapolis, Indiana). Mice were maintained in quarantine for at least one week. Following quarantine, initial blood counts were taken and mice were tagged and shaved in preparation for tumor cell inoculation. Following this, mice were allowed to rest another week before initiation of experiments. Mice were housed in autoclaved cages, and were maintained in an air-conditioned, specific pathogen-free animal room regulated at a temperature of 21° to 23°C and a relative humidity of 50% to 60%. The 12-hour lighting cycle began with lights on at 8 AM. Mice were given commercial rodent chow and water ad libitum. All experiments in this study were approved by the Institutional Animal Care and Use Committee at BATTS Laboratories (Northridge, CA).

### Cell lines

The EL4 murine lymphoma (EL4) (ATCC #TIB-39) and the Lewis murine lung cancer (LL) cell lines (ATCC# CRL-1642) were purchased from the American Tissue Culture Collection (ATCC). Cells were grown in Dulbecco's Modified Eagle's Medium with 10% horse serum (EL4) or 10% FBS (LL). Prior to inoculation, tumor cells were counted, centrifuged, washed with phosphate buffered saline (PBS), and resuspended in PBS. C57BL/6 female mice were inoculated with 100,000 EL4 tumor cells, and C57BL/6 male mice were inoculated with 100,000 LL tumor cells by implanting (subcutaneous injection) the tumor cells on the backs of the mice.

### Cytokines

Murine recombinant IL-12 and murine recombinant G-CSF were purchased from PeproTech, Inc. (Rocky Hill, NJ), and prepared according to manufacturer's instructions. Lyophilized mrIL-12 was dissolved in PBS and lyophilized mrG-CSF was dissolved in water. All subsequent dilutions were prepared using PBS. Cytokine or vehicle injections were administered in volumes of 100 μl.

### Chemotherapy

Cyclophosphamide (Cytoxan) was purchased from Bristol Myers-Squibb. The dry powder (500 mg) was dissolved in saline for injection, according to manufacturer's instructions.

### Study design

For both radiation and chemotherapy studies, experiments were initiated by first inoculating mice with tumor cells, as described above. For the radiation studies, mice were inoculated with tumor cells and 2 days later received total body irradiation (625 rad) using the dual, opposed sources of a cesium 137 irradiator (Atomic Energy of Canada, Model: γ-cell 40). For chemotherapy studies with cyclophosphamide, mice were inoculated with tumor cells in the same manner as was done for the radiation studies; however, for the chemotherapy studies palpable tumors were allowed to develop. In the case of the EL4 lymphoma model, large tumors, about 12 mm in one dimension were allowed to develop before initiation of cytokine treatments and chemotherapy. For the Lewis lung cancer model, the experiment was initiated when tumors were about 5 mm in one dimension. Pre-treatment doses of IL-12 were administered 24 hours before radiation or chemotherapy, while post treatment doses of IL-12 or G-CSF were given within 2 hours following radiation or 48 hours following chemotherapy. Peripheral blood cell counts were determined 2 days before radiation treatment and about every 3–4 days following radiation or chemotherapy. Vehicle, IL-12 and G-CSF were administered intravenously. Cyclophosphamide (Cytoxan) was administered intraperitoneally. All experimental groups for radiation studies contained 7 mice and for the chemotherapy studies there were 8 animals in each group. Peripheral blood samples were obtained from the retro-orbital plexus of anesthetized mice using 75-mm capillary tubes (Fisher Scientific, Pittsburg, PA (#22-362-574)). The blood sample was then transferred from the capillary tube to a EDTA-coated blood collection tube (Sarstedt, Numbrecht, Germany (#20.1278.100)). Complete blood cell counts were performed using the Hemavet 850 FS (Drew Scientific, Oxford, CT).

### Assessment of bone marrow cellularity

At the end of the radiation and chemotherapy experiments bone marrow samples were taken from surviving mice. Femurs were removed from IL-12-treated and control animals (vehicle and G-CSF) and fixed in 10% formalin buffer. The femurs were embedded in TissuePrep 2 paraffin wax for micro-section at 5 μM and routine hematoxylin & eosin (HE) staining was performed. Slides were examined microscopically (Nikon E CLPSE E 8000, Camera: Diagnostic Model 2.2.1 with Spot RT software V3.5) and percent cellularity was visually quantified.

### Tumor measurements

Tumor growth was generally assessed by biweekly measurement of tumor diameters with a Vernier caliper. Two formulas for the calculation of tumor volumes were used. For the radiation studies, the diameter of the tumor was measured and the following formula was used V = 4/3πr^3^. For chemotherapy studies, two dimensions of tumor were measured and the following formula was used: Tumor weight = Tumor Volume = d^2 ^× D/2, where d and D are the shortest and longest diameters, respectively. Treated animals were checked daily for treatment toxicity/mortality. The percentage of tumor growth inhibition was calculated in either case as %T/C = 100 × (mean tumor volume of treated group/mean tumor volume of control group) [37,38]. For clinical studies, the National Cancer Institute (NCI) standard for significance in cytoreductive data is defined as %T/C < 50% [38]. Thus, in accordance with NCI standards, if the difference between the treated and control groups yield a reduction in tumor volumes that is less than 0.5, the results are considered significant and the agent is considered "active" in the tumor studied in clinical trials. For the preclinical data presented, the animal number was too small to yield statistical significance given the high variance in tumor growth. Consequently, we used the NCI clinical standard as a means to evaluate the differences in reduction in tumor volumes as a function of treatment group for the results presented in both the radiation and chemotherapy studies. Thus, the use of the term "significant" in the presentation of the tumor data does not denote statistical significance, although "a T/C in the 42–50% range would usually meet statistical significance at the 5% level" [38]. Chi square analysis of tumor incidence was also employed as variability in tumor size precluded ANOVA.

### Comparative microarray analyses

Aliquots of total RNA from the EL4 and Lewis lung cancer cell lines were used for microarray processing on the Affymetrix Genechip^® ^Mouse Genome 430 2.0 Array (Santa Clara, CA). Total RNA from each cell line was extracted using TRIZOL (Sigma, St. Louis, MO). Double-stranded cDNA was synthesized from total RNA. To identify secreted factors, the microarray results were further evaluated for the presence of the following properties known to be associated with secreted/soluble proteins: 1) the presence of a predicted signal peptide and no TMD indicative of the gene product being processed via the secretory protein pathway, 2) the presence of a predicted single transmembrane domain (TMD), with or without the presence of a predicted signal peptide, indicative of a gene product that is membrane-bound, but capable of being released from the cell membrane, i.e., processed so that the extracellular domain becomes soluble, and 3) the presence of one or more predicted O- or N- glycosylation sites in gene products that do not possess either a predicted signal peptide or a single TMD. The algorithms used to determine the signal peptide properties and TMD properties are SignalP and TMHMM, respectively, both available at the Center for Biological Sequence Analysis (CBS) prediction server [39]. Predictive algorithms for the presence of glycosylation sites were also obtained at the CBS server. These algorithms are called NetNGlyc and NetOGlyc for the prediction of N- and O- glycosylation sites, respectively.

### Statistical analyses

The overall strategy for the statistical analyses is as follows. First, the sample size (n) chosen for these studies was determined from previous studies that showed that a sample size of at least 7 was required to reliably detect changes in blood variables among the groups at an α level of .05 and a power of 0.8. In the various analyses, missing values (*e.g*. some monocyte values were 0 or missing) and a few outliers in the data set were replaced by the case mean for the particular blood variable. Excluding the outliers and replacing the missing values, however, did not appreciably affect the results. The first analysis performed in each experiment was an overall repeated measures MANOVA (RMANOVA). RMANOVA determined whether the treatment groups could be distinguished over time in terms of a set of five dependent variables, *i.e*., the blood measurements for neutrophils, lymphocytes, monocytes, red blood cells and platelets. The tumor model variable (lymphoma vs. lung cancer) was included as a between groups variable in these analyses, as well as in subsequent analyses, if the model variable or interaction with the other variables was significant; otherwise it was dropped. If the RMANOVA was significant with a main effect of Group (i.e., the groups could be distinguished with this set of variables), and a Group*Blood Cell Type interaction (i.e., group differences varied across blood measures) or Group*Blood Cell Type*Day interaction (i.e., group differences varied across blood measures and over days), subsequent MANOVAs (or RMANOVAs) were performed for each of the five dependent blood measures with days treated as separate dependent variables (MANOVA) or replicates (RMANOVA) and groups as defined above. Next, if these MANOVAs yielded a significant main effect of Group (or if RMANOVAs yielded Gp*Day interactions), then ANOVAS were performed on individual days for individual blood variables. If a significant main effect of Group was found in the daily ANOVAs, post hoc Tukey tests were performed to determine which groups were different on the particular day. The results from the Tukey tests are reported in terms of p-values below.

## Results

### Precise scheduling of low dose IL-12 significantly improves pancytopenia in sublethally irradiated tumor-bearing mice

In previous studies we found that IL-12 administered at a dose of 100 ng (5 μg/kg) via intravenous injection was the optimal dose for rescuing lethally irradiated normal mice [36]. We also found that this dose of IL-12, administered only once, 24 hours before or 1 hour after lethal radiation conferred a significant survival benefit to lethally irradiated mice [36]. Specifically, about 92% and 80% of the mice were rescued from a lethal dose of radiation (1000 rad), as compared to control mice, when a single, low dose of IL-12 was administered before or after radiation, respectively [36]. Further, the IL-12 lethal radiation survival effect was obtained using the lowest effective, single dose ever used [36], as compared to other cytokines, such as IL-1 [17], SCF [18] and TPO [19] (See Table 1). It is also important to emphasize that IL-12 is effective when administered either before or after radiation, which was not the case for IL-1, SCF or TPO, as these cytokines could only rescue mice when administered either before (IL-1), twice before and once after (SCF), or after (TPO) a lethal dose of radiation (See Table 1). Apparently, among these cytokines, only IL-12 and TPO can rescue mice when administered as single doses after a lethal dose of radiation. IL-12, however, appears to be superior to TPO in its ability to confer survival from lethal radiation, as IL-12 is effective at lower doses than TPO and is also effective at higher radiation doses (See Table 1).

**Table 1:** Lethal Radiation Rescue in Mice for Early-acting Cytokines

**Cytokine**	**Dose**	**Dosing Schedule**	**% Survival**	**Reference**
IL-1β	3000 ng	single dose, -20 hr	100 (n = 10)	17
SCF	2000 ng	-20 h, -2 h, +4 h	100 (n = 20)	18
TPO	300 ng	single dose, +2 h	90 (n = 4–8)*	19
TPO	300 ng	single dose, +2 h	70 (n = 20)**	19
IL-12	100 ng	single dose, -24 h	91 (n = 38)***	36
IL-12	100 ng	single dose, +1 h	78 (n = 35)***	36

*radiation dose was 800 rad for C57BL/6 mice; **radiation dose was 900 rad for C57Bl/6; ***radiation dose was 1000 rad for C57BL/6 mice

In the present studies, we examined the effects of various IL-12 dosing schedules on hematopoietic recovery from sublethal radiation in tumor-bearing mice using the optimal dose (100 ng or 5 μg/kg)) found in our previous lethal radiation studies [36]. We compared the effects of IL-12 (100 ng/total dose) on hematopoietic recovery using three dosing schedules, specifically: 1) IL-12 pre-dosing at 24 hours before radiation, 2) IL-12 post-dosing at 2 hours after radiation, and 3) a split dose of IL-12, *i.e*., pre-post dosing using 50 ng given both 24 hours before and 2 hours after radiation. Experimental controls were vehicle (PBS) or G-CSF. G-CSF was administered at in a 1 μg (50 μg/kg) dose intravenously 2 hours after radiation. Since G-CSF is a FDA-approved cytokine for hematopoietic recovery that is widely used clinically, it was used in these studies as a positive control. G-CSF was not administered before radiation in these tumor-bearing mice, as previous experimentation showed that pre-dosing with G-CSF resulted in premature death of the mice (data not shown). Further, G-CSF is never given before radiation or chemotherapy in the clinical setting.

In addition, mice in all treatment groups also received four subsequent, non-repeated, doses of vehicle, G-CSF or IL-12 about every 3–4 days following radiation. For the G-CSF treatment groups, the same dose as the initial dose (1 μg/dose) was maintained for subsequent dosing following radiation. However, for the IL-12 treatment groups, mice received a lower dose of IL-12 (30 ng/dose) than the dose originally administered. It is also notable that the total dose of G-CSF used in these experiments is about 23 times higher than the dose of IL-12 on a weight basis (about 90 times higher on a molar basis).

**Figure 1:**
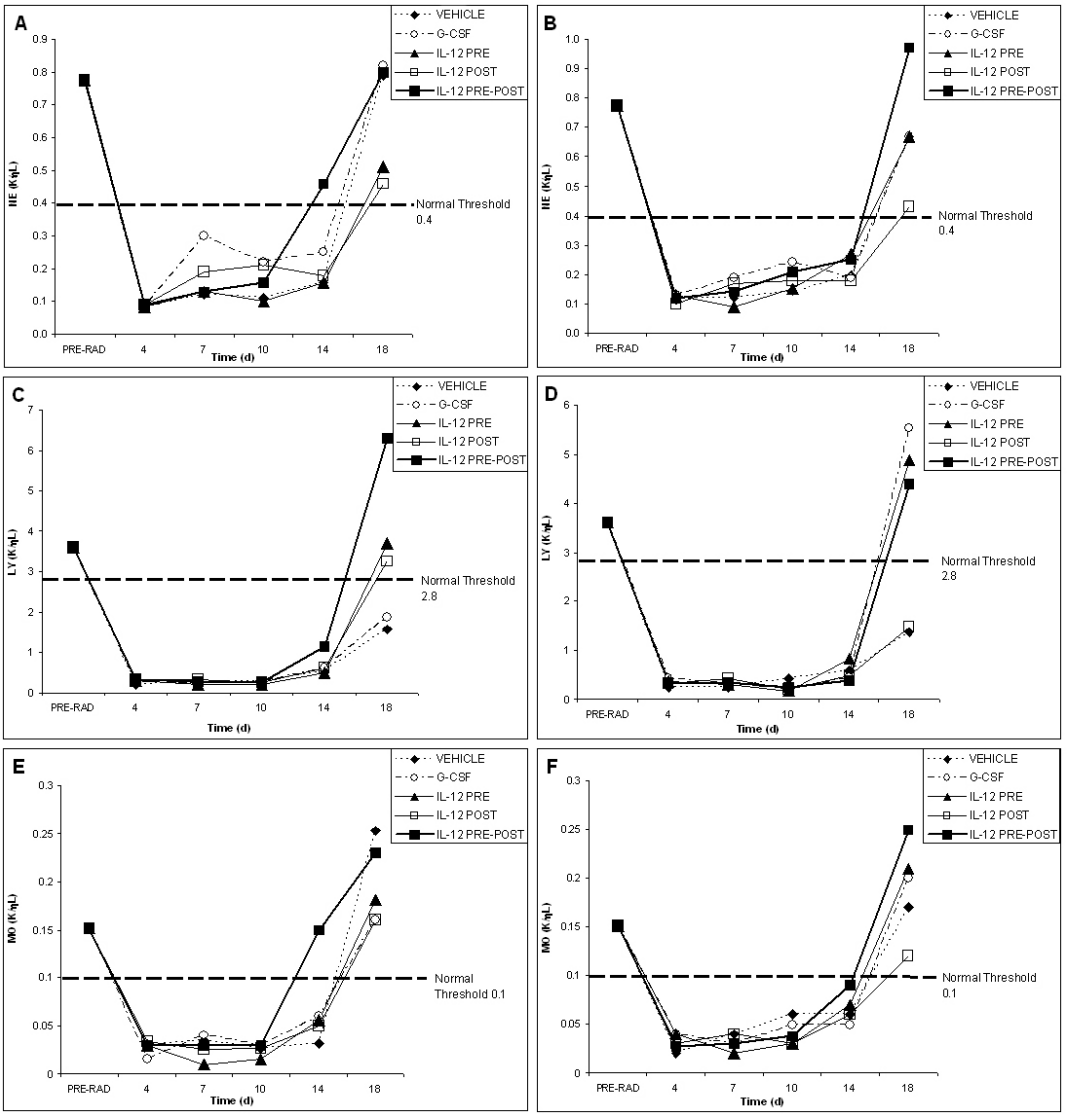
**White blood cell recovery profiles for vehicle, G-CSF, IL-12 pre-only, IL-12 post-only and IL-12 pre-post treatment groups following radiation (625 rad)**. White blood cell recovery profiles are shown for the EL4 tumor model (left; A, C and E) and the Lewis lung cancer model (right; B, D, and F) for neutrophils (A and B), lymphocytes (C and D) and monocytes (E and F). Pre-radiation blood values reflect an average of all animals in both groups (70 mice) before radiation and tumor inoculation. The normal threshold values for neutrophil, lymphocyte and monocyte counts in C57BL/6 mice are indicated. Following the initial dose of vehicle or cytokine, 4 subsequent doses were given every 3–4 days following radiation. IL-12 treated mice received a total dose (5 single doses) of 220 ng, whereas G-CSF treated mice received a total dose (5 single doses) of 5 μg.

**Figure 2:**
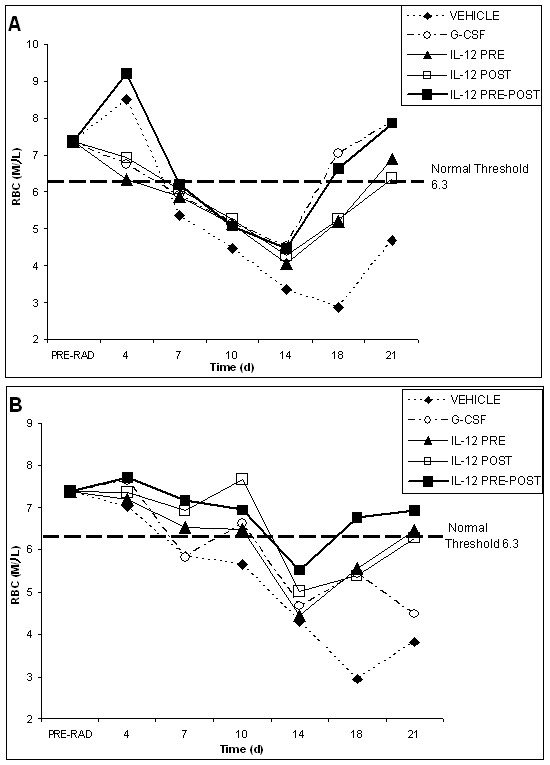
**Red blood cell recovery profiles for vehicle, G-CSF, IL-12 pre-only, IL-12 post-only and IL-12 pre-post treatment groups following radiation (625 rad)**. Red blood cell recovery profiles are shown for the EL4 tumor model in (A) and the Lewis lung cancer model in (B). Pre-radiation blood values reflect an average of all animals in both groups (70 mice) before radiation and tumor inoculation. The normal threshold value for murine red blood cell counts is indicated. Following the initial dose of vehicle or cytokine, 4 subsequent doses were given every 3–4 days following radiation. IL-12 treated mice received a total dose (5 single doses) of 220 ng, whereas G-CSF treated mice received a total dose (5 single doses) of 5 μg.

**Figure 3:**
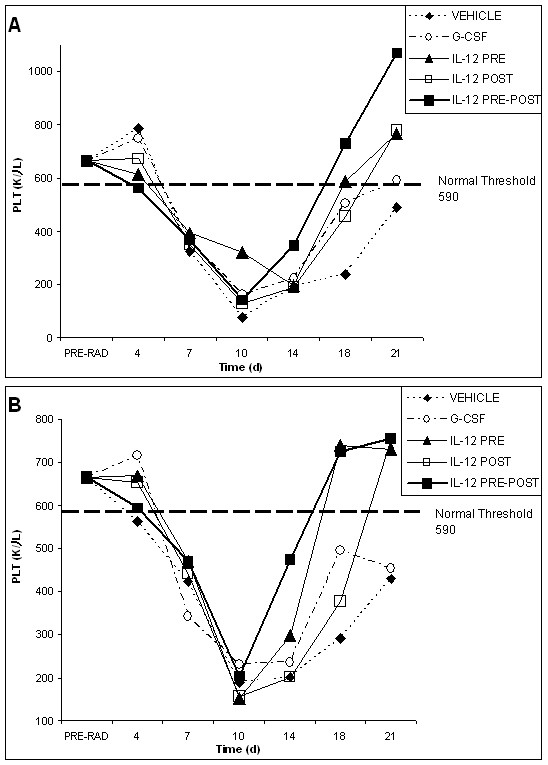
**Platelet recovery profiles for vehicle, G-CSF, IL-12 pre-only, IL-12 post-only and IL-12 pre-post treatment groups following radiation (625 rad)**. Platelet recovery profiles are shown for the EL4 tumor model in (A) and the Lewis lung cancer model in (B). Pre-radiation blood values reflect an average of all animals in both groups (70 mice) before radiation and tumor inoculation. The normal threshold value for murine platelet counts is indicated. Following the initial dose of vehicle or cytokine, 4 subsequent doses were given every 3–4 days following radiation. IL-12 treated mice received a total dose (5 single doses) of 220 ng, whereas G-CSF treated mice received a total dose (5 single doses) of 5 μg.

The recovery profiles of various peripheral blood cell counts from the myelosuppressive effects of radiation are shown in Figures 1, 2, 3 for both tumor models. For the recovery profiles shown in the figures, the threshold value for each blood cell type is indicated. The threshold values for neutrophils, lymphocytes and monoctyes represent the lower range of normal blood values as sampled from about 200 C57BL/6 mice. The threshold values given for red blood cells and platelets represent the lower range of normal values for all murine sub-species (provided by Drew Scientific for use with the Hemavet 850FS).

Statistical analyses using repeated measures multivariate analysis of variance (RMANOVA) with treatment group and tumor model as between variables, blood cell types as the dependent variables, and days as the replicate, produced a significant main effect of group (Gp, p < .001) indicating the treatment groups could be discriminated on the basis of these independent variables. Furthermore, a significant Gp*Day or Gp*Day*Blood Cell Type interaction justified subsequent MANOVAs or RMANOVAs on each blood cell variable. No significant main effect or interaction with tumor model was observed and that between variable was dropped from further analyses. In additional data file 1, Table 3 presents the significant results in these MANOVAs, subsequent ANOVAs on individual days following significant Gp*Day interactions and Tukey tests for between group differences on those days indicated by the preceding ANOVAs. Also in additional data file 1, Table 5 presents the relevant Tukey tests for the radiation studies.

Figure 1(A–F) comparatively depicts the white blood cell (wbc) recovery profiles for neutrophil (ne), lymphocyte (ly) and monocyte (mo), respectively, following radiation for both tumor models. All three subtypes of wbc groups reached very early (at day 4 post radiation) nadirs for all treatment groups, as compared to red blood cell (rbc) and platelet (plt) nadirs (shown in Figures 2 and 3).

Figure 1(A–B) shows the recovery of neutrophil counts following radiation for the two tumor models. Figures 1A (EL4) and 1B (LL) show that although the neutrophil counts are severely depressed following radiation (~10–15% of normal values), neutrophil recovery is comparatively facile, as all treatment groups, including the vehicle control, attain normal neutrophil counts by day 18 in both tumor models. Of note in the EL4 lymphoma model is the IL-12 pre-post treatment group, which reached normal neutrophil levels early at day 14 (Fig. 1A) (p < 0.05 as compared to vehicle; all p values given herein are derived from posthoc Tukey tests as described in the Methods)). In fact, in the EL4 tumor model, early IL-12 pre-post facilitated recovery is noted for all wbc groups. For the Lewis lung cancer model (Fig. 1B), the IL-12 pre-post treatment group exhibited early recovery from about day 10, as compared to the vehicle control. Further, for the LL model, only the IL-12 pre-post treatment group yielded statistically significant differences in neutrophil recovery on day 14, as compared to the vehicle control in both tumor models (p < 0.05). G-CSF is used clinically in cancer patients to promote neutrophil recovery. Thus, it was surprisingly that the G-CSF-facilitated neutrophil recovery appeared to start early on day 7 (p < 0.001 as compared to vehicle), but then declined in both tumor models between days 10 and 14.

Figure 1(C–D) shows the lymphocyte recovery profiles following radiation for both tumor models. As compared to the other wbc, the decline in lymphocyte counts is the most severe. Less than 10% of the normal lymphocyte counts remain following radiation at days 4 to about day 14. This highly depressed lymphocyte state is apparent for all groups, except for the IL-12 pre-post treatment group in both tumor models, where recovery started at about day 10. For the EL4 lymphoma model, the IL-12 pre-post group showed early lymphocyte recovery starting at about day 10 and attained above threshold lymphocyte counts at day 18, whereas G-CSF and vehicle groups do not even reach normal threshold values by day 18. For the Lewis lung cancer model, both IL-12 pre-post and G-CSF treatment groups attain normal lymphocyte counts by day 18. However, an analysis of lymphocyte recovery for the various treatment groups revealed that only the IL-12 pre-post treatment group yielded statistically significant lymphocyte recovery in both tumor models at day 18, as compared with the vehicle control (p < 0.05).

The monocyte recovery profiles are shown in Figure 1(E–F) for both tumor models. Like neutrophils, monocyte recovery is relatively facile, as monocyte counts also reached normal levels by day 18 for all treatment groups in both tumor models. Thus, there are no significant differences among the various treatment groups for monocytes on day 18. The IL-12 pre-post treatment group, however, was the only group that showed statistically significant early monocyte recovery on day 14 in both tumor models (p < 0.05).

Figure 2(A–B) shows the red blood cell (rbc) recovery profile for both tumor models. First, it is notable that rbc require a relatively long time to reach the nadir in either tumor model. This phenomenon is expected and can be attributed to the relatively long half-life of rbc. From inspection of Figure 2, it is also notable that the rbc nadir following radiation for the vehicle is observed at day 18 in both tumor models, whereas the rbc nadir is sharply attenuated and shifted to an earlier time point, namely day 14, for all cytokine treatment groups in both model systems. The rbc recovery for the vehicle group started to occur after day 18, but never fully recovered in the 21 day observation period for both tumor models. The rbc recovery profile in both tumor models indicates that although this blood cell group may be the least sensitive to radiation, the red blood cells are slow to repopulate following radiation.

The statistical analysis for rbc recovery profiles, shown in Figure 2, reveal that at the cytokine-facilitated nadir on day 14, the IL-12 pre-post treatment group is the only group to yield a statistically significant decrease in myelosuppression, as compared to the vehicle group (p < 0.05). On day 18, all cytokine treatment groups are statistically different from the vehicle group (p < 0.001), despite the fact that not all treatment groups have yet attained normal rbc levels. For the lymphoma model, both the IL-12 pre-post and the G-CSF treatment groups reached normal rbc levels on day 18. For the Lewis lung cancer model, however, only the IL-12 pre-post group attained normal rbc levels on day 18. It is also notable that for the Lewis lung cancer model, the IL-12 pre-post treatment group yielded only a short period of slight anemia. On day 21 post radiation, all cytokine groups are statistically different from the vehicle group (p < 0.001 for IL-12 pre-post, p < 0.005 for IL-12 pre-only, and p < .05 for both IL-12 post-only and G-CSF). Overall, it is notable that only the IL-12 pre-post treatment group yielded significant early (day 7) red blood cell recovery in both tumor models (P = 0.05 as compared to vehicle), as well as consistently superior overall red blood cell recovery from the myelosuppressive effects of radiation.

Figure 3(A–B) shows the platelet recovery profile for both tumor models following radiation. The platelet profile for all treatment groups generally showed a sharp nadir at about 10 days. However, for the Lewis lung cancer model, the nadir for the G-CSF treatment group was protracted, lasting from day 10 to day 14. Also for the EL4 lymphoma model, the nadir of the IL-12 pre-only treatment group is significantly elevated at day 10 and shifted to a later time point at day 14. For the EL4 lymphoma model, all cytokine treatment groups show a slight attenuation of the nadir for platelet counts at day 10 as compared to the vehicle group. For the Lewis lung cancer model, the IL-12 pre-post and G-CSF treatment groups show marginal attenuation of the platelet nadir as compared to the vehicle control. It is notable that a significant attenuation of the platelet nadir was observed in our radiation studies of normal, non-tumor bearing mice [36], thus revealing a possible effect of the tumor microenvironment on platelet counts. For the vehicle control, the platelet counts decline to about 10% of normal on day 10 for the EL4 lymphoma model and about 30% of normal on day 10 for the Lewis lung cancer model. These differences may be attributed to the differences in the microenvironment of the two tumor models.

Following the nadir in platelet counts at day 10, generally all cytokine treatment groups start to show recovery of platelet counts. Remarkably though, the IL-12 pre-post treatment group shows the earliest and sharpest rise in platelet counts starting on about day 14. Moreover, this early recovery of platelet counts for the IL-12 pre-post group is statistically significant in both tumor models, as compared to both the G-CSF treatment group (p = 0.001) and the vehicle control (p < 0.001). On day 18, all cytokine treatment groups, except the IL-12 post-only group (marginal), show statistically significant platelet recovery, as compared to the vehicle control in both tumor models (p < 0.001 for IL-12 pre-only and pre-post groups and p = 0.001 for G-CSF). Also on day 18, the IL-12 pre-post and pre-only treatment groups showed statistically significant recovery as compared to G-CSF (p < 0.001 and p < 0.05, respectively). Further, only the IL-12 pre-post and pre-only treatment groups attained normal platelet levels on day 18 in both tumor models. On day 21, all IL-12 treatment groups yielded statistically significant platelet recovery as compared to the vehicle group (p < 0.01 for the IL-12 pre-only group, p < 0.005 for the IL-12 post-only group and p < 0.001 for the IL-12 pre-post group). Also as compared to the G-CSF treatment group on day 21, all IL-12 treatment groups yielded statistically significant platelet recovery (p < 0.001, p = 0.05 and p > 0.05 for the pre-post, pre-only and post-only treatment groups, respectively). It is notable that although a statistically significant effect on platelet recovery was found in our previous radiation studies in normal mice [36], the observed IL-12 effect on platelet recovery in tumor-bearing mice appears to be more potent, perhaps due to differences in the dosing schedules of IL-12, as well as the higher radiation dose, used in these studies.

Overall, these radiation studies show that the pre-post split dose of IL-12 yielded consistent, statistically significant hematopoietic recovery from the effects of radiation for every blood group tested, as compared to the G-CSF and vehicle controls, whereas the two other dosing schedules were less effective in our radiation studies, but similarly effective in our chemotherapy studies as discussed below.

### Low dose IL-12 provides a concomitant reduction in tumor volumes to hematopoietic recovery from sublethal radiation, as compared to radiation alone, in tumor-bearing mice

**Figure 4:**
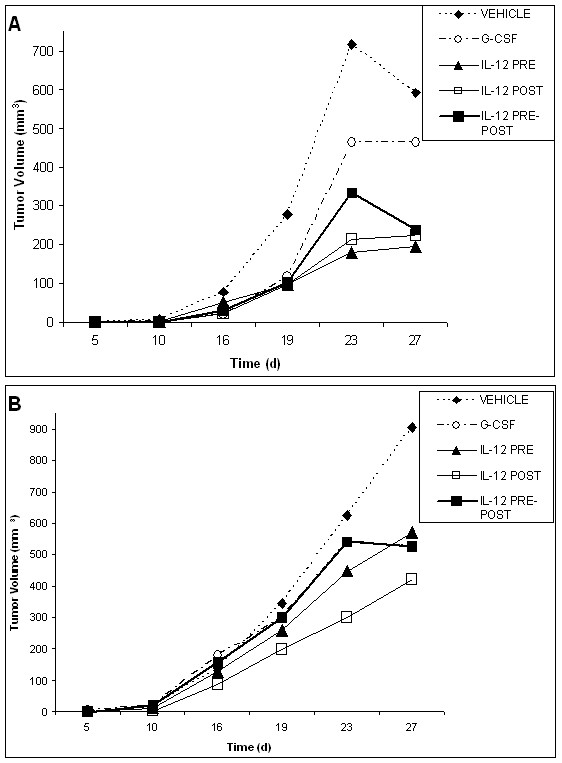
**Relative changes in tumor volumes for vehicle, G-CSF, IL-12 pre-only, IL-12 post-only and IL-12 pre-post treatment groups following radiation**. Changes in tumor volumes over the course of the experiment are shown for the EL4 lymphoma tumor model (A) and the Lewis Lung cancer tumor model (B). Mice in both tumor models were given 625 rad at day 1. Mice in the EL4 lymphoma tumor model were given a second dose of radiation on day 22 (560 rad), and mice in the Lewis Lung cancer tumor model also were given a second dose of radiation on day 22 (750 rad) following the initial radiation dose. Tumor measurements were made on the days indicated by measuring the tumor diameter and converting this measurement into tumor volume using a spherical volume formula. In the EL4 lymphoma model, all IL-12 treatment groups significantly reduced tumor growth (%T/C < 50%) as compared with the PBS and G-CSF controls at the end point of tumor volume evaluation. In the Lewis lung cancer model, only the IL-12 post-only treatment group gave a significant reduction in tumor growth (%T/C < 50%) at the end point of tumor volume evaluation. The change in the slope of the curve for days 19–23 as compared to days 23–27, which occurs after the second radiation, may indicate a radio-sensitizing effect of the indicated treatments.

The antitumor properties of IL-12 in mice are well-known and replete in the literature. Although the present studies are focused on the less well-known hematological properties of IL-12, it is significant that IL-12 treatment reduced tumor volumes in both tumor models under conditions appropriate for facile hematopoietic recovery. The concomitant antitumor effects of IL-12 as an adjuvant to radiation therapy are shown in Figures 4A and 4B for the EL4 lymphoma and the Lewis lung cancer models, respectively. In these studies, an assessment of the change in tumor volumes was made for all treatment groups about every 3–6 days for 27 days post-radiation. On day 22, all mice received a second dose of radiation, so that changes in tumor volumes following a second radiation dose could be observed.

All cytokine treatment groups exhibited an overall reduction in tumor volume at the end point of the study for both tumor models, as compared to the vehicle control group. For the lymphoma model, however, treatment with IL-12 (pre-post, pre-only or post-only) significantly reduced tumor growth (%T/C < 50%) as compared to the vehicle control, whereas the results for G-CSF were not significant. Interestingly, all treatment groups appeared to show some radiosensitivity to the second dose of radiation in the lymphoma model. In the Lewis lung cancer model, the G-CSF and IL-12 treatment groups showed essentially the same reduction in tumor volume, as compared to the vehicle control. Although for the Lewis lung cancer model, all cytokine treatment groups yielded a reduction of tumor growth, as compared to the vehicle control, only the IL-12 pre-post and G-CSF treatment groups showed signs of radiosensitivity to the second dose of radiation. Also in the Lewis lung cancer model, only the IL-12 post-only treatment group yielded a significant reduction in tumor volume (%T/C < 50%), as compared to the vehicle control.

We also employed a non-parametric analysis of tumor incidence among the various treatment groups across both tumor models. We utilized the chi square test to examine relative tumor incidence because of the high variance in tumor size within groups, possibly due to the very aggressive nature of the tumor models. We found that generally tumor incidence was lower for all cytokine-treated groups, as compared to the vehicle (chi square = 12.3, p = .01). Furthermore, this was particularly true for the IL-12 post-only treatment group on all days in both tumor models, as compared to the vehicle control (chi square = 6.5, p < 0.01).

### Low dose IL-12 facilitates hematopoietic recovery following chemotherapy in tumor-bearing mice

The drug chosen for use in our chemotherapy studies was cyclophosphamide, as this drug is often used clinically and is specifically used in the treatment of both lymphoma and lung cancer (personal communication from D. Douer). All treatment groups were maintained as in our radiation studies with some modifications, as indicated below.

The statistical analyses for these chemotherapy studies (RMANOVA analyses), however, indicated differences in tumor models; thus, all statistical analyses were done separately for each model. The presence of tumor model differences for the chemotherapy studies should be contrasted with our radiation studies, where no model differences were found (RMANOVA analyses), and therefore, data from the two tumor models were combined for the statistical analyses. Model differences in these chemotherapy studies are likely due to the differences in the initiating tumor volumes for these late stage lymphoma and lung cancer models, as discussed below.

From preliminary studies, we ascertained that hematopoietic recovery following cyclophosphamide without the use of an adjuvant therapy (vehicle alone) was much more facile as compared to radiation. Following chemotherapy using cyclophosphamide, we found that full recovery of most peripheral blood cell counts took days, rather than several weeks as observed in our radiation studies. The shorter time for hematopoietic recovery following one round of chemotherapy was expected, as most chemotherapies, and particularly cyclophosphamide, are not marrow-damaging, and consequently, leave the stem cell compartment relatively intact. Given this fact, we decided to use a later stage tumor model for our chemotherapy studies. Our radiation studies necessitated the use of earlier stage tumor models because of the time frame of blood recovery relative to tumor growth. Of note is the fact that both the EL4 lymphoma and Lewis lung cancer cell lines yield highly aggressive tumor models, whereby mice without treatment usually die within 2–3 weeks.

Therefore, for the chemotherapy studies, mice were inoculated with tumor cell lines in the same manner as performed in the radiation studies, but the experimental treatment protocols were instead initiated when tumors were palpable in both tumor models. For the EL4 model the experimental treatment protocols, however, were initiated when tumor volumes were very large, *i.e*., with an average tumor volume of about 500 mm^3^. Tumors were allowed to grow large in the EL4 model because in preliminary studies we found that the EL4 tumors disappeared in all treatment groups, including the vehicle control, following cyclophosphamide treatment. Apparently, for the EL4 lymphoma murine model, as is true in clinical lymphoma, cyclophosphamide provides potent antitumor effects (personal communication from D. Douer). Consequently, the dose of cyclophosphamide used was 250 mg/kg for the EL4 lymphoma model and 225 mg/kg for the Lewis lung cancer model, given the very large, pretreatment tumor volumes for the EL4 model.

Another modification to our chemotherapy protocol was the time period for post cytokine administration. Preliminary studies revealed that the optimal time to administer cytokines post-chemotherapy was 48 hours after cyclophosphamide treatment, as compared to our radiation studies in which cytokines were administered 2 hours following radiation. Moreover, this is generally the time period for post-chemotherapy administration of G-CSF clinically (personal communication from D. Douer). Pre-treatment with IL-12, however, was still performed under the same schedule as in our radiation studies, at 24 hour before chemotherapy. The cytokine treatment dosages used were also changed in the chemotherapy studies. The pre-dose for both the IL-12 pre-post and pre-only treatment groups was 50 ng. The post dose for the IL-12 pre-post group was 100 ng, while the IL-12 post-only dose was 150 ng. Therefore, for the IL-12 pre-only group the total dose was 50 ng, whereas for the IL-12 pre-post and post-only treatment groups the total dose was 150 ng. For G-CSF the dose was increased to 1.5 μg. Unlike our radiation studies, however, no subsequent doses of cytokines or vehicle were administered following the 48 hour post-cyclophosphamide cytokine treatment in these chemotherapy studies.

Repeated measures multivariate analysis of variance (RMANOVA) with treatment group and tumor model as between variables, blood cell types as the dependent variables, and days as the replicate produced a significant main effect of group (Gp, p < .001) indicating the treatment groups could be discriminated on the basis of these dependent variables. Furthermore, a significant Gp*Day or Gp*Day*Blood Cell Type interaction justified subsequent MANOVAs or RMANOVAs on each blood cell variable. Here we did see a significant interaction with tumor model (Gp*Model*Day; p < .001, or Gp*Model*Day*Blood Cell Type, p < .001) which necessitated separate analyses for the two tumor models. In additional data file 1, Tables 4A and 4B present the significant results separately for the two models, specifically the MANOVAs, subsequent ANOVAs on individual days following significant Gp*Day interactions and Tukey tests for between group differences on those days indicated by the preceding ANOVAs. Also in additional data file 1, Table 6 presents the relevant Tukey tests for the chemotherapy studies.

**Figure 5:**
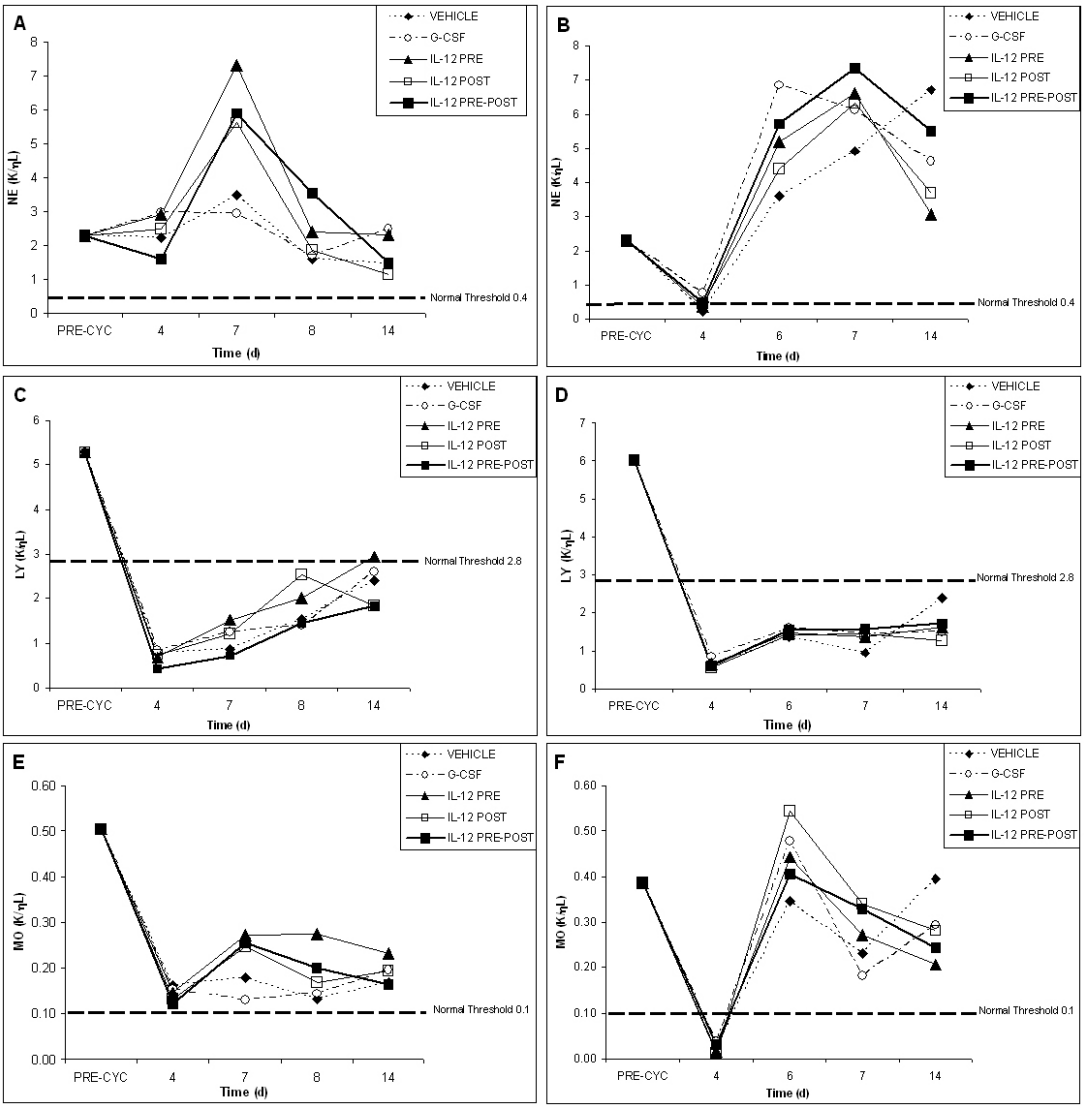
**White blood cell recovery profiles for vehicle, G-CSF, IL-12 pre-only, IL-12 post-only and IL-12 pre-post treatment groups following chemotherapy**. White blood cell recovery profiles are shown for the EL4 tumor model (left; A, C and E) and the Lewis lung cancer model (right; B, D, and F) for neutrophils (A and B), lymphocytes (C and D) and monocytes (E and F). Pre-chemotherapy blood values reflect an average of all animals in both groups (80 mice) before cyclophosphamide and tumor inoculation. The normal threshold values for neutrophil, lymphocyte and monocyte counts in C57BL/6 mice are indicated. EL4 mice received a cyclophosphamide dose of 250 mg/kg and the LL mice received a cyclophosphamide dose of 225 mg/kg. IL-12 treated mice received a total dose of 50 ng (pre-only) and 150 ng (post-only and pre-post (pre-post dose was split 50 ng before and 100 ng after cyclophosphamide)), whereas G-CSF treated mice received a total dose 1.5 μg.

**Figure 6:**
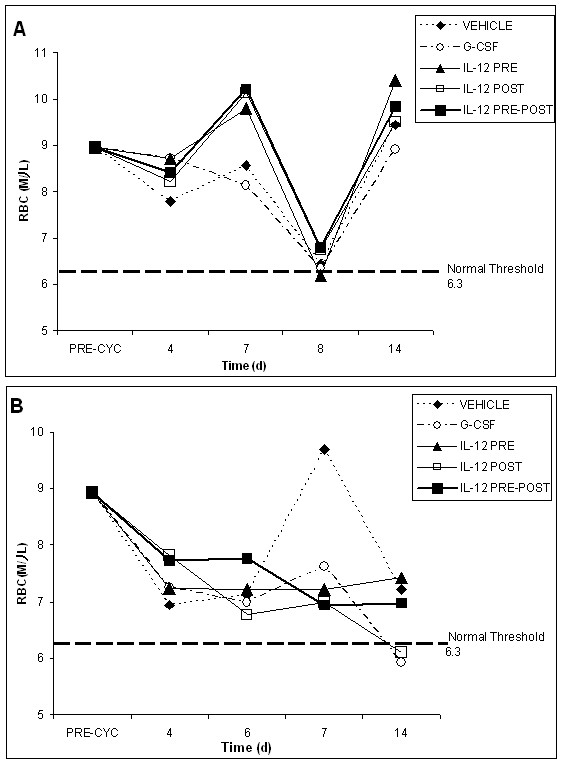
**Red blood cell recovery profiles for vehicle, G-CSF, IL-12 pre-only, IL-12 post-only and IL-12 pre-post treatment groups following chemotherapy**. Red blood cell recovery profiles are shown for the EL4 tumor model in (A) and the Lewis lung cancer model in (B). Pre-chemotherapy blood values reflect an average of all animals in both groups (80 mice) before cyclophosphamide and tumor inoculation. The normal threshold value for murine red blood cell counts is indicated. EL4 mice received a cyclophosphamide dose of 250 mg/kg and the LL mice received a cyclophosphamide dose of 225 mg/kg. IL-12 treated mice received a total dose of 50 ng (pre-only) and 150 ng (post-only and pre-post (pre-post dose was split 50 ng before and 100 ng after cyclophosphamide)), whereas G-CSF treated mice received a total dose 1.5 μg.

**Figure 7:**
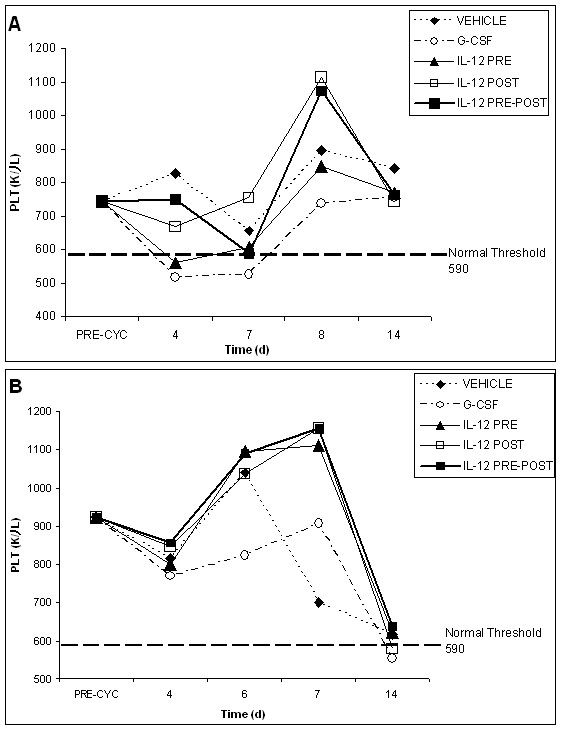
**Platelet recovery profiles for vehicle, G-CSF, IL-12 pre-only, IL-12 post-only and IL-12 pre-post treatment groups following chemotherapy **. Platelet recovery profiles are shown for the EL4 tumor model in (A) and the Lewis lung cancer model in (B). Pre-chemotherapy blood values reflect an average of all animals in both groups (80 mice) before cyclophosphamide and tumor inoculation. The normal threshold value for murine platelet counts is indicated. EL4 mice received a cyclophosphamide dose of 250 mg/kg and the LL mice received a cyclophosphamide dose of 225 mg/kg. IL-12 treated mice received a total dose of 50 ng (pre-only) and 150 ng (post-only and pre-post (pre-post dose was split 50 ng before and 100 ng after cyclophosphamide)), whereas G-CSF treated mice received a total dose 1.5 μg.

Figures 5, 6, 7 show the hematopoietic recovery profiles for relevant blood cell groups. The results reveal that despite the high dose of cyclophosphamide used in these experiments prolonged myelosuppression was not generally observed. Only the lymphocyte population remained substantially suppressed during the 14 day post treatment observation period, especially in the Lewis lung cancer model. For all other blood cell counts, the peripheral counts were either normal by day 14, or did not go below normal for the entire observation period. For instance, although all wbc counts were highly depressed following cyclophosphamide treatment, the neutrophil and monocyte counts were observed to quickly "bounce-up" to normal or above normal levels within about 3 days following the nadir. This "bounce-up" of certain wbc counts is also observed clinically with cyclophosphamide treatment [40] Although changes in the rbc and platelet counts were apparent during the observation period, these peripheral blood counts generally did not go below normal values for any of the treatment groups, including the vehicle control during the observation period. Overall these results are consistent with the marrow-sparing properties of cyclophosphamide, whereby the cytotoxicity of the drug generally affects only mature cells, and especially mature cells of the white blood cell lineage [personal communication from D. Douer].

Upon inspection of the wbc recovery profiles, shown in Figure 5(A–F), a significant difference can be noted in the extent of myelosuppression at the nadir for treatment groups in the EL4 model, as compared to the Lewis lung cancer model. Overall, the nadir observed at day 4 for neutrophils and monocytes is less depressed, *i.e*., higher, in the EL4 lymphoma model as compared with the lung cancer model, whereas the lymphocytes are more similarly depressed in the two models. Moreover the 4 day "nadir" for neutrophils in the EL4 model is not clearly defined and appears to show a functional dependence on treatment group.

Figure 5(A–B) shows the neutrophil recovery profile following cyclophosphamide treatment for both tumor models. As discussed above, the neutrophil recovery profile for the EL4 model, shown in Figure 5A, does not show a clear nadir for all treatment groups at day 4. These results can be compared to the sharper nadir observed for neutrophil counts at day 4 in the Lewis lung cancer model (ANOVA p < .01). This differential behavior is likely due to the differences in the initiating tumor volumes in the two models. Generally in both the EL4 and Lewis lung cancer tumor models, we observed a rise in neutrophil counts with increasing tumor volumes. This observation has been made in numerous murine [41-43] and even in human cancers [44,45], and has been attributed to a rise in the level of tumor-secreted factors with increasing tumor burden.

Another significant fact to note in relation to both the neutrophil and monocyte profiles, shown in Figures 5(A–B) and 5(E–F), is that cyclophosphamide is known to mobilize progenitor cells from the bone marrow into peripheral blood. Thus, the data suggest that the facile recovery of neutrophil and monocyte counts following cyclophosphamide treatment is likely due to the ability of this drug to mobilize neutrophils and monocytes to the periphery in the tumor models. The mobilization effect of cyclophosphamide can be inferred by the facile recovery to above normal neutrophil counts on day 7 post-chemotherapy for the vehicle group in both tumor models, which occurs just a few days following the nadir.

In the EL4 lymphoma model (Fig. 5A), neutrophil recovery for all IL-12 treatment groups is quite similar throughout the 14 day observation period following cyclophosphamide treatment. Moreover for the EL4 tumor model, all the IL-12 treatment groups showed greater neutrophil counts on the peak recovery day, *i.e*., day 7, as compared to the G-CSF and vehicle controls, which appeared to be quite similar. In fact, all IL-12 treatment groups in the EL4 model show about a 3-fold increase in neutrophil counts above baseline levels and about a 2-fold increase above the vehicle control at day 7. This high "bounce-up" of the neutrophil counts with IL-12 treatment is likely due to an additional mobilizing effect of IL-12, which was described previously [35]. The lack of a "bounce-up" of the neutrophil counts for the G-CSF treatment group in the EL4 model is surprising, as G-CSF is used clinically to mobilize progenitor cells for transplantation in the treatment of hematopoietic malignancies [40]. This lack of mobilization effect of G-CSF may be due to use of a single administration of G-CSF in these studies, whereas the clinical mobilization of progenitor cells by G-CSF generally requires about 5 daily, or twice daily, administrations of the cytokine for effective mobilization. The apparent facile neutrophil mobilization effect of IL-12, using just a single, low dose, suggests the possible superiority of IL-12, as compared to G-CSF, for the mobilization of progenitor cells for use in hematopoietic cellular transplantation. At the end of the 14 day observation period, all treatment groups returned to normal levels in the EL4 lymphoma model, as compared to the Lewis lung cancer model, which is discussed below.

**Figure 8:**
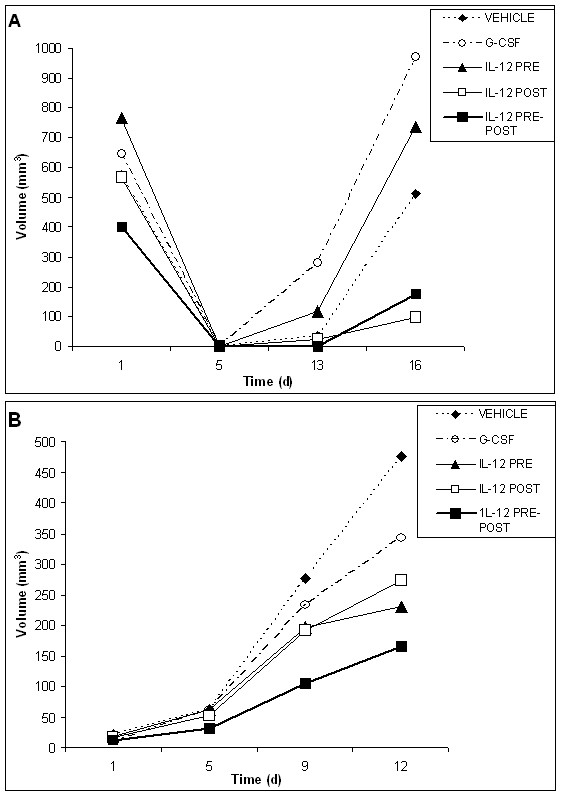
**Relative changes in tumor volumes for vehicle, G-CSF, IL-12 pre-only, IL-12 post-only and IL-12 pre-post treatment groups following chemotherapy**. Changes in tumor volumes over the course of the experiment are shown for the EL4 lymphoma tumor model (A) and the Lewis Lung cancer tumor model (B). IL-12 treatment significantly reduces tumor growth following cyclophosphamide treatment in late stage tumor models. In the EL4 tumor model, treatment with IL-12 pre-post or IL-12 post-only significantly reduced tumor growth (%T/C < 50%), as compared to both the G-CSF vehicle control groups at the end point of tumor growth assessments. In the Lewis lung cancer model, the IL-12 pre-post and IL-12 pre-only treatment groups yielded a significant reduction in tumor growth (%T/C < 50%), as compared to both the G-CSF and vehicle control groups at the end point of tumor growth assessments. These tumor models represent a late stage cancers, as compared with the models used for the radiation studies because tumors were apparent at the start of the treatments consisting of cyclophosphamide and cytokine therapy. In the case of the lymphoma model, therapy was initiated when the tumors were very large (about 500 mm^3^).

Neutrophil recovery profiles for the various treatment groups in the Lewis lung cancer model (fig 5B) appeared to be very different as compared to the EL4 model. In the LL tumor model, all cytokine treatment groups exhibit about a 3-fold "bounce-up" of the neutrophil counts, as compared to baseline levels, at about 7 days post-chemotherapy (ANOVA p < .07, marginal, at day 6). This "bounce up" in neutrophil counts is apparently due to the ability of both IL-12 and G-CSF to provide a mobilization effect over and above the primary therapy (vehicle group) in the lung cancer model. Another notable difference in the lung cancer model, as compared to the EL4 model, is that neutrophil counts do not return to normal levels at the end of the 14 day observation period, especially for the vehicle control. This effect is likely due to the differences in tumor progression in the two tumor models, as shown in Figure 8. For the lung cancer model tumors are fully present by day 14 post chemotherapy, thus yielding a concomitant rise in neutrophil counts, whereas for the EL4 tumor model, tumors are just starting to reappear at about day 13 following cyclophosphamide therapy. The differential data from the two tumor models underscore the importance of assessing hematopoietic recovery in tumor-bearing hosts.

The lymphocyte recovery profile is shown for both tumor models in Figure 5(C–D). The recovery profile in both models has a nadir at day 4, as do all the wbc counts following chemotherapy. As previously stated, the lymphocyte counts do not return to baseline levels during the 14 day observation period, but in the EL4 model, the lymphocytes counts approached normal threshold levels. There are no significant differences in the recovery profiles for the IL-12, G-CSF and vehicle treatment groups in both tumor models. Although lymphocyte recovery was observed in our radiation studies, the recovery appeared later at about day 18.

The monocyte recovery profile, shown in Figure 5(E–F), also displays a nadir at day 4. It is notable that the baseline monocyte counts are higher in the EL4 model, as compared to the Lewis lung cancer model, most likely due to the relatively high tumor burden at the initiation of treatment for the EL4 model. Further, the recovery profile for the EL4 lymphoma model is relatively flat as compared to the Lewis lung cancer model. For the EL4 model, monocyte counts for all treatment groups are in the normal range throughout the 14 day observation period, even at the nadir. The relatively high monocyte count at the nadir for the EL4 tumor model coincides with the disappearance of the tumors at day 5, as shown in Figure 8A below. Moreover for the EL4 model, although there does not appear to be any significant differences in monocyte recovery as a function of treatment group, overall monocyte counts are higher following the nadir for all IL-12 treatment groups as compared to the G-CSF and vehicle controls. For the Lewis lung cancer model, however, there is a sharp nadir at day 4 with a "bounce-up" recovery at day 6 post-chemotherapy. Although there are observable differences in monocyte counts as a function of treatment group at day 6, these differences are slight with the G-CSF and IL-12 treatment groups yielding higher monocytes counts as compared to the vehicle group. Once again, the differences in blood cell recovery for monocytes in the two tumor models point to the importance of assessing hematopoietic recovery in tumor bearing hosts.

Figure 6(A–B) shows the rbc recovery profile for both tumor models following chemotherapy. It is notable that a single dose of cyclophosphamide treatment does not cause anemia in these murine model systems, even at the high doses utilized in these studies. Thus, there is no apparent nadir in both model tumor models, although the red blood cells are decreased at about day 8 in the EL4 lymphoma model and about day 4 in the Lewis lung cancer model in all treatment groups. For the EL4 lymphoma model, IL-12 treatment groups maintain the red blood cell counts at a higher overall level, as compared to the G-CSF and vehicle controls during the 14 day observation period. In the Lewis lung cancer model, a significant increase in rbc counts was observed for the IL-12 pre-post and post-only treatment groups on day 4, as compared to the vehicle control (p < 0.05 and p = 0.01, respectively). Overall, significant differences among the treatment groups were not observed in the LL model, except that the vehicle group appears to uniquely show a spike in rbc counts on day 7.

The platelet recovery profile following cyclophosphamide treatment is shown in Figure 7(A–B) for both tumor models. As observed for rbc counts, the platelet counts do not generally go below normal during the 14 day observation period, except for the G-CSF treatment group and the IL-12 pre-only group in the EL4 lymphoma model. In the EL4 lymphoma model, a slight depression in platelet counts is observed at day 7, but only the G-CSF treatment group yielded below normal platelet counts. Interestingly for the IL-12 pre-post and post treatment groups a "bounce-up" of the platelet counts was observed at day 8 in the EL4 lymphoma model. For the Lewis lung cancer model, a "bounce-up" of platelet counts was also observed on day 7 for all cytokine treatment groups, but only for all IL-12 treatment groups did the rise in platelet counts go above pre-chemotherapy values. Moreover in the Lewis lung cancer model, all IL-12 treatment groups were equally effective in raising platelet counts above baseline values at day 7, as compared to the G-CSF and vehicle controls. This is particularly remarkable for the IL-12 pre-only treatment group where the rise in platelet counts in the lung cancer model occurred at a dose of only 50 ng, which is 1/30 of the dose of G-CSF (1.5 μg) on a weight basis (and less than 1/100 of the dose of G-CSF on a molar basis).

Further, the effect of IL-12 on platelet counts is statistically significant in both tumor models. On day 8 in the lymphoma model, the IL-12 post-only treatment groups yielded a significant rise in platelet counts as compared to the vehicle control (p < 0.05) Also as compared to G-CSF on day 8, the IL-12 post-only and pre-post treatment groups produced a statistically significant rise in platelets (p < 0.001) in the EL4 model.

In the Lewis lung cancer model, all cytokine treatment groups yielded a statistically significant rise in platelet counts as compared to the vehicle control on day 7 (p = 0.01 for G-CSF, and p < 0.001 for all IL-12 treatment groups). Also on day 6 and day 7, IL-12 treatment groups showed statistically significant differences in the rise in platelet counts as compared to G-CSF (day 6: p = 0.01 for the IL-12 pre-only and pre-post groups; day 7: p = 0.01 for the IL-12 pre-only group and p < 0.001 for both the IL-12 post-only and IL-12 pre-post groups). For the Lewis lung cancer model, it is remarkable that even for the IL-12 pre-only treatment group, where IL-12 was administered at a dose of 50 ng, only once, at 24 hours before chemotherapy, the rise in platelet counts was statistically significant as compared to both the vehicle and G-CSF controls. Overall, given that cyclophosphamide treatment did not yield a significant depression in platelet counts in these chemotherapy experiments, the significant rise in platelet counts as a function of IL-12 treatment suggests a possible role for IL-12 in thrombopoiesis.

### Low dose IL-12 provides a concomitant reduction in tumor volumes to hematopoietic recovery from chemotherapy, as compared to the chemotherapy alone, in tumor-bearing mice

We also assessed the antitumor effects of IL-12 in conjunction with cyclophosphamide treatment. These results are shown in Figure 8(A–B) for both tumor models. As stated above, for these chemotherapy studies a later stage tumor model was utilized as compared to the tumor model used in the radiation studies. A later stage tumor model could be utilized in these chemotherapy studies because the time frame of hematopoieitic recovery relative to tumor growth was compatible, *i.e*., a shorter time period for hematopoietic recovery following cyclophosphamide treatment was needed as compared to recovery from radiation.

In these chemotherapy studies, IL-12 treatment in conjunction with cyclophosphamide generally yielded a significant reduction in tumor volume (%T/C < 50%) at the end of the observation period in both tumor models, as compared to the primary therapy alone (vehicle group). Specifically for the EL4 model, both the IL-12 pre-post and post-only groups yielded a significant reduction in tumor volume (%T/C < 50%) at the end of the 16 day observation period. However, for the lung cancer model, the IL-12 pre-post (split dose of 50 ng/100 ng) and pre-only treatment groups (50 ng) yielded a significant reduction in tumor volume (%T/C < 50%) at the end of the 12 day observation period. G-CSF treatment did not provide significant tumor reduction in either tumor model, but did yield an overall non-significant reduction in tumor volume in the lung cancer model. However in the lymphoma model, tumor growth (volume) was observed to be much greater for the G-CSF group than for the vehicle control. Also, in the lymphoma model, the IL-12 pre-only treatment group yielded tumor volumes greater than the vehicle control. These results for the G-CSF and the IL-12 pre-only treatment groups in the lymphoma model are likely related to the very large initiating tumor volumes utilized in this tumor model.

Another significant aspect of the IL-12 antitumor effect of IL-12 in the Lewis lung cancer model is that IL-12 in conjunction with cyclophosphamide yielded a significant antitumor response over and above the primary therapy alone despite the fact this cell line is non-immunogenic. Previous reports of the effect of IL-12 on the Lewis lung cancer tumor model showed that IL-12 had no antitumor effect with this non-immunogenic tumor [46]. Thus, our results for the IL-12 pre-post treatment group in this non-immunogenic tumor, where %T/C is about 34%, suggest that the combination of chemotherapy and IL-12 altered the immunogenicity of this non-immunogenic lung cancer model.

A second round of chemotherapy and cytokine treatments was initiated in the lymphoma model to further assess tumor development in mice whose tumor diameters were less than 20 mm. Cyclophosphamide and adjuvant cytokine treatments were performed in the same manner as described above. The percentage of mice with tumor diameters less than 20 mm at the initiation of the second round of treatments is shown in Table 2. At this point in the study there was only one death in the G-CSF group.

**Table 2:** EL4 lymphoma mice with tumor volumes < 20 mm at start of the second round of chemotherapy (cyclophosphamide)

**Treatment Group**	**% mice (< 20 mm)**
Vehicle	37.5
G-CSF	37.5
IL-12 pre	37.5
IL-12 post	62.5
IL-12 pre/post	75.0

**Figure 9:**
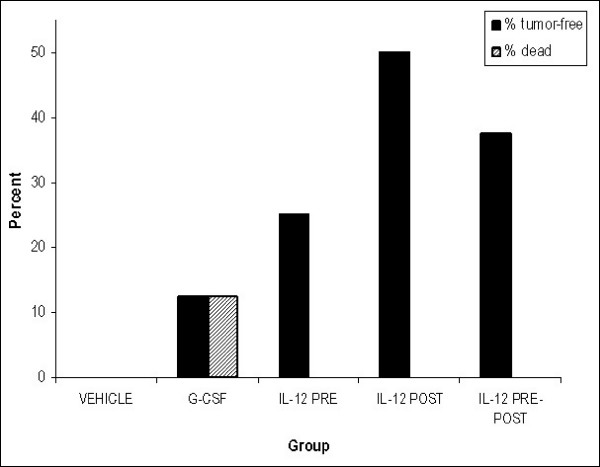
**Percentage of mice in the vehicle, G-CSF, IL-12 pre-only, IL-12 post-only and IL-12 pre-post treatment groups that were tumor-free following the second round of cyclophosphamide treatment**. The percentage of mice in the lymphoma study that remained tumor-free at the end of the second round of cyclophosphamide (225 mg/kg), which was 5 weeks from the first round of cyclophosphamide treatment and 7 weeks after tumor inoculation. No mice in the vehicle groups were tumor-free. There was one death in the G-CSF treatment group.

Several observations are notable. In the second round of chemotherapy, the tumors did not disappear in mice with visible tumors, as they did in the first chemotherapy round. Thus, in the second round of cyclophosphamide, there was an apparent tolerance to the cytotoxic agent, as compared to the first round of chemotherapy in these murine model systems. Tolerance to cyclophosphamide is also observed for human cancers [47]. The percentage of mice in the lymphoma study that remained tumor-free at the end of the second round of chemotherapy, which was 5 weeks from the first round of cyclophosphamide treatment and 7 weeks after tumor inoculation, are shown in Figure 9. Interestingly, for the IL-12 pre-post and post-only groups 37.5% and 50% of the mice, respectively, were tumor-free at the termination of the experiment, whereas no control mice were found to be tumor-free. Thus, IL-12 treatment coupled with cyclophosphamide was a very effective treatment in this very aggressive lymphoma model, yielding antitumor effects well over the very potent effects of the primary therapy alone. It is notable that no mice from the Lewis lung cancer model were tumor-free at any time during, or at the end of, the experiment.

### Assessment of bone marrow cellularity following radiation or chemotherapy treatment regimens

**Figure 10:**
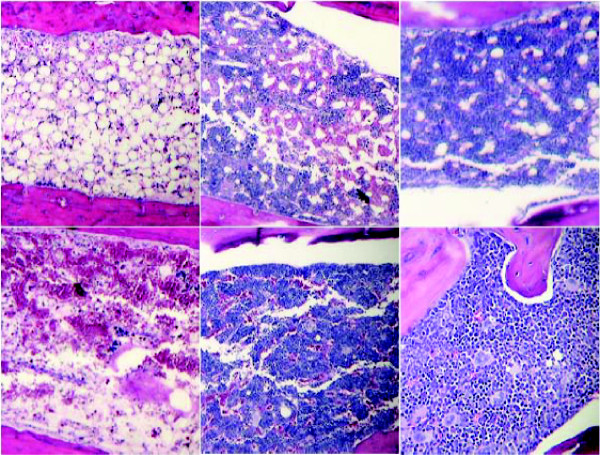
**Pathology of bone marrow for the vehicle, G-CSF, IL-12 pre-only, IL-12 post-only and IL-12 pre-post treatment groups following radiation**. Bone marrow specimens for the EL4 lymphoma model (top) and the Lewis lung cancer model (bottom) are shown. For the EL4 model, vehicle (top, left), G-CSF (top, middle) and IL-12 pre-post (top, right) are shown. For the Lewis lung cancer model (bottom), vehicle (bottom, left) and IL-12 pre-only (bottom, middle) are shown. No G-CSF treated mice survived the second radiation dose. A normal (untreated) control bone marrow specimen is also shown (bottom, right).

Pathological changes in the bone marrow for various treatment groups after two rounds of radiation in the two tumor models are shown in Figure 10. For the EL4 lymphoma model (top), bone marrow cellularity was assessed for mice surviving 40 days following two doses of sublethal radiation (625 rad on day 0 and 560 rad on day 22). Thus, for this tumor model, bone marrow specimens were taken 18 days following the second radiation dose. Inspection of Figure 10 (top) clearly shows differences in bone marrow cellularity for the vehicle, G-CSF and IL-12 pre-post treatment groups, which were assessed to be at 10%, 40% and 90% cellularity, respectively, as compared to a normal control (lower right). For the Lewis lung cancer model, bone marrow cellularity, as shown in Figure 10 (bottom), was assessed 30 days following the two doses of sublethal radiation (625 rad on day 0 and 750 rad on day 22). For this model, bone marrow specimens were taken 8 days following the second radiation dose. In the Lewis lung cancer model, however, there were no surviving mice from the G-CSF treatment group; consequently no comparative assessment of bone marrow cellularity could be made for this group. Inspection of Figure 10 (bottom) clearly shows differences in bone marrow cellularity for the vehicle as compared to the IL-12 pre-only group, which were assessed at 10% and 60% cellularity, respectively as compared to a normal control (lower right).

Bone marrow cellularity was also assessed following cyclophosphamide treatment for all treatment groups in both tumor models. No significant changes in bone marrow cellularity as a function of treatment group were observed, as all treatment groups exhibited bone marrow cellularity at 90–100% of the normal control. This finding is consistent with the relative marrow-sparing properties of cyclophosphamide treatment, as compared with the marrow-damaging properties of radiation.

Pathological analysis was performed for other organs following radiation and chemotherapy. Specifically, liver, spleen, intestinal, lymph, and lung tissue were assessed. No increased pathological effects of IL-12, as compared to the vehicle control, were found. In fact, typically the organs from IL-12 treated animals appeared as healthy as, or in better condition than, the control group.

### Comparative microarray analyses reveal different profiles of tumor-associated secreted factors for the EL4 as compared to the LL tumor cell lines

The incorporation of the tumor model into the experimental protocol for the assessment of IL-12-facilitated hematopoietic recovery was undertaken due to previous reports that described the difficulty of obtaining hematopoietic recovery in tumor-bearing hosts [48,49]. It has been suggested that factors secreted from tumors affect the stem cell compartment leading to a depletion of the stem cell pool [48]. Other reports demonstrated that tumor-secreted factors can lead to immunosuppression [50-52].

The data from our radiation and chemotherapy studies revealed notable differences in behavior of the various treatment groups in the two tumor models. Also, granulocytosis, exhibited as above-normal neutrophil counts, generally accompanied late stage tumor growth in the tumor models, especially in the lung cancer model. These observations prompted us to assess possible differences in the tumor-associated secreted factors from the EL4 lymphoma and Lewis lung cancer cell lines, as one possible source of the differential hematopoietic recovery. Specific knowledge of the tumor-associated secreted factors is important to the understanding of the tumor models, as well as human cancers, as these factors may contribute to autocrine and/or paracrine effects that can affect hematopoietic recovery as well as tumor growth.

In additional data file 2, Tables 7A and 7B list the gene expression levels for some of the highly expressed gene products possibly secreted into circulation in the EL4 lymphoma and Lewis lung cancer model systems, respectively. Interestingly, differential secreted factor patterns are observed for the different cell lines, which may be related to the differential hematopoietic recovery, as well as tumor growth, in the two model systems.

Of the secreted factors listed in Tables 7A and 7B (additional data file 2), it is remarkable that many have been reported to play a role in cancer. Some notable tumor-associated factors found in both the EL4 and Lewis lung cancer cell lines are hepatoma-derived growth factor (HDGF), vascular endothelial growth factor A (VEGFA) and platelet-derived growth factor C (PDGFC), transforming growth factor beta (TGFβ), and marcrophage colony stimulating factor (CSF1). Further, many of the factors found expressed in these tumor cell lines display angiogenic properties, and as such are generally suspects in the promotion of cancer. Notable tumor-associated factors that appear to be highly expressed only in the Lewis lung cancer cell line that are also known to be involved in cancer, are osteopontin, kit ligand (stem cell factor), chemokine (CXC) ligand 1, pleiotropin, fibroblast growth factor 7, amphiregulin and epiregulin.

A notable factor expressed in both the EL4 and Lewis lung cancer cell lines is marcrophage colony stimulating factor (CSF-1). The putative secretion of this factor in the two tumor models may be responsible for the observed granulocyotosis (high neutrophil count) as a function of high tumor burden, as colony stimulating factors have been frequently implicated in the granulocytosis found in murine [43] and human cancers [44,45]. Interestingly, inspection of the Tables 7A and 7B (additional data file 2) reveals that CSF-1 is more highly expressed in the Lewis lung cancer cell line, as compared to the EL4 cell line. These data provide a putative basis for our observation that granulocytosis (neutrophilia) was more apparent in the Lewis lung cancer model, as compared to the EL4 model, during the radiation and chemotherapy studies.

The gene expression data listed in Tables 7A and 7B (additional data file 2), as compared to normal tissue counterparts found at the Symatlas tissue expression database [53] hosted by the Genomics Institute of the Novartis Research Foundation (GNF) reveal that the Lewis lung cancer model appears to generate many more up-regulated tumor-associated factors than the EL4 lymphoma cell line. Although both cell lines used in these studies are highly aggressive, the Lewis lung cancer appeared to be more deadly. Mice in the lung cancer model generally died earlier as compared to the lymphoma model. It is interesting to speculate that the tumor-associated factors expressed in the Lewis lung cancer cell line are related to the aggressiveness of this murine cancer, and possibly, that these factors may also be important to the refractory nature of human lung cancer.

## Discussion

IL-12 has been extensively studied for nearly two decades for its role in immunity and for its antitumor properties [24]. A role for IL-12 in hematopoiesis was noted sporadically in the early literature [31-35], but seems to have been forgotten, as this role was overshadowed by the role of IL-12 in immunity. Consequently, the role of IL-12 as a cytokine that can connect, or bridge, the immune and hematological systems, acting at both the level of mature and primitive hematopoietic cells, has not been appreciated. Our present studies elucidate the role of IL-12 as a hematological adjuvant cancer therapy, and as such, begin to shed light on its role as an important bridging cytokine capable of linking the immune and hematological systems.

Our initial studies of the role of IL-12 in hematopoiesis started with a demonstration of its potent ability to rescue the hematopoietic system from the deleterious effects of lethal radiation [36]. With an appreciation of this fundamental and potent role of IL-12 in hematopoiesis, we postulated a clinical role for IL-12 as a hematological adjuvant cancer therapy. In this role, we envisioned IL-12 being used as an adjuvant to be used along with standard chemotherapy and radiation therapy regimens. We further hypothesized that in this role IL-12 would provide effective hematopoietic recovery from the hematological toxicity of the primary therapy, and concomitantly, provide antitumor effects over and above those of the primary therapy. The present studies were undertaken in an effort to find preclinical support for this hypothesis.

We found that indeed IL-12 could provide effective and facile hematopoietic recovery from the effects of radiation (625 rad) or chemotherapy (cyclophosphamide) at single, low doses in two tumor model systems, while still providing concomitant antitumor responses. Importantly, these effects were obtained without any apparent toxicity. The fact that low doses were effective in our studies is significant because if relatively high, or repeated, doses are required to obtain an effect using cytokine therapy, generally toxicity will result. This is especially true for early acting cytokines, like IL-12, IL-1, SCF and TPO. Our studies also show that as compared to G-CSF, which is widely used clinically, IL-12 yields more consistent and potent multilineage hematopoietic recovery, especially from the marrow-damaging effects of radiation, while still providing significant antitumor responses in the two tumor models studied.

In the present studies, our goal was to assess hematopoietic recovery following myelosuppression in a system that would more closely resemble the clinical setting. Thus, we chose to assess hematopoietic recovery in tumor-bearing hosts. Tumor-bearing hosts serve many purposes in the studies: 1) they provide a rigorous test of the ability of IL-12 to promote hematopoietic recovery, as previous reports described the difficulty in obtaining blood recovery in tumor-bearing hosts [47,48] especially following the marrow-damaging effect of radiation [47], 2) they more closely predict the behavior of IL-12 in cancer patients, and 3) they simultaneously allow an evaluation of the dual hematopoietic and antitumor properties of IL-12.

To better understand the tumor models chosen for study, we performed comparative microarray analyses of the EL4 lymphoma and Lewis lung cancer cell lines. From these analyses, we compared the gene expression levels for tumor-associated secreted factors that are expressed in these cell lines, and therefore, are possibly secreted into circulation in the EL4 lymphoma and Lewis lung models. Interestingly, we found some similar, but many different, genes expressed related to tumor-associated secreted factors in the different cell lines. The differential expression profiles for these putative secreted factors may be one source of the observed differences in hematopoietic recovery for the various cytokine treatment groups, as well as tumor growth, in the two models utilized in our studies. Another notable source of these observed differences is the sex of the mice, as the EL4 lymphoma model utilized female mice and the Lewis lung cancer model utilized male mice. Our statistical analyses, however, revealed that there was no model difference in our radiation studies utilizing an early tumor model, but did find differences due to model in the late stage tumor model utilized in our chemotherapy studies. This fact suggests that the tumor model, rather than sex, was the dominant variable responsible for the observed differences in hematopoietic recovery in the two model systems.

For the radiation studies, IL-12 pre-post treatment at the single dose of 50 ng, both before and after radiation, yielded statistically significant recovery of neutrophils, lymphocytes, monocytes, red blood cells and platelets, as compared to the vehicle control. Interestingly, for these radiation studies in tumor-bearing mice, the IL-12-faciliated neutrophil recovery appeared to be equal, if not superior, to G-CSF, which is a cytokine used clinically for its neutrophil-promoting effects. Moreover, this effect was facilitated by IL-12 at about 1/90 the dose of G-CSF (on a molar basis). Overall what stands out from these radiation studies is the ability of the IL-12 pre-post treatment group to consistently yield superior hematopoietic recovery of every major blood cell group in both tumor models, as compared to both the vehicle and G-CSF controls.

Consistent accelerated hematopoietic recovery effects of IL-12 were also observed in the relatively more marrow-sparing regimen of cyclophosphamide treatment in both tumor models. Using cyclophosphamide to induce myelosuppression we found that only white blood cells were generally affected. Of the white blood cells, only the neutrophils and monocytes clearly showed a cytokine-facilitated recovery during the 14 day observation period. In the chemotherapy studies, the hematopoietic recovery performance of G-CSF in the EL4 lymphoma model, which was initiated with a high tumor burden, was surprisingly poor. Interestingly, in this late stage lymphoma model, the ability of G-CSF to facilitate neutrophil and monocyte recovery was similar to that of the vehicle control. On the other hand, all IL-12 treatment groups yielded enhanced neutrophil and monocyte recovery as compared to the G-CSF and vehicle controls in the EL4 lymphoma model. For the Lewis lung cancer model, which was initiated at a lower tumor burden, IL-12 and G-CSF showed a similar ability to yield enhanced neutrophil and monocyte recovery above that of the vehicle control. These results underscore the profound role of the tumor microenviroment in cytokine-facilitated hematopoietic recovery following myelosuppression.

An interesting and unexpected result, shown both in the radiation and chemotherapy studies, was the highly consistent and statistically significant effect of IL-12 on platelet recovery. In our radiation studies of the EL4 lymphoma model, the IL-12 pre-post treatment group was the only group to attain above normal threshold values for platelets at day 18 following radiation. At 21 days post radiation, all IL-12 treatment groups were above normal threshold platelet values. For the Lewis lung cancer model, both the IL-12 pre-post and pre-only treatment groups attained full platelet recovery at day 18 post-radiation. Once again at 21 days post-radiation, all IL-12 treatment groups yielded full platelet recovery in the Lewis lung cancer model, as observed in the lymphoma model. Furthermore, neither G-CSF nor the vehicle group was able to achieve full recovery of platelet counts, *i.e*., above normal threshold values, during the 21 day observation period in either tumor model.

Surprisingly, even in our chemotherapy studies, where the platelet counts were not generally affected by treatment with cyclophosphamide, a statistically significant IL-12-facilitated "bounce-up" of platelet counts was observed in both tumor models. For the EL4 lymphoma model, the platelet counts for the IL-12 post-treatment group were statistically different from the vehicle group on day 8. Also in the EL4 lymphoma model, the IL-12 pre-post and post-only groups yielded statistically significant differences in platelet counts as compared to G-CSF on days 4, 7 and 8 post-chemotherapy (See supplemental Table 6). For the Lewis lung cancer model, all IL-12 treatment groups similarly yielded a "bounce-up" in platelet counts on day 7, including the IL-12 pre-only group where the total dose of IL-12 was only 50 ng. These results for the Lewis lung cancer model following cyclophosphamide treatment were highly significant as compared to both the vehicle and the G-CSF treatment groups.

The platelet effect of IL-12 in our chemotherapy studies, where the platelets rise above the normal baseline, also has been observed with TPO administration to normal mice [54,55]. This effect of IL-12 on platelet counts suggests a direct role of IL-12 in thrombopoiesis, including megakaryopoiesis. Moreover, the IL-12-faciliated platelet recovery effect following sublethal radiation observed in the present studies, and in our previous studies in normal mice, is similar to the effect of peg-rHuMGDF (at similar dosages), a TPO derivative, following sublethal radiation in normal mice [20]. Peg-rHuMGDF, like TPO, however, is not being pursued clinically due to adverse effects in Phase I clinical trials. Taken together, the results from our radiation and chemotherapy studies in tumor-bearing hosts, as well as our previous studies in normal mice [36] suggest that IL-12 plays a role in thrombopoiesis. To our knowledge, this is the first report of the thrombopoietic properties of IL-12. The remarkable effect of IL-12 on platelet recovery following myelosuppressive therapy may be of clinical importance, as there is currently no available drug that can facilitate platelet recovery following myelosuppression. Studies to elucidate the role of IL-12 in thrombopoiesis and megakaryopoiesis are underway in our laboratory.

Overall, the results presented, coupled with our previous studies of the lethal radiation effect of IL-12, strongly suggest that IL-12 is a hematopoietic stem cell factor. Further, the consistency of the IL-12-faciliated hematopoietic recovery in both tumor models following radiation or chemotherapy, even under conditions of high tumor burden, suggest that IL-12 is an early-acting hematopoietic factor. Studies underway in our laboratory have identified a sub-population of hematopoietic stem cells (HSC) that exists in murine and human bone marrow that are marked by the presence of the IL-12 receptor (IL-12R^+^) on lineage negative cells. Moreover, these IL-12R^+ ^HSC are responsive to administration of the IL-12 ligand. We speculate, therefore, that the IL-12 receptor is a positive functional marker defining HSC. We also have found that exogenous administration of the IL-12 ligand can expand, *in vivo*, HSC marked by the IL-12 receptor. These HSC expansion properties of IL-12 provide a basis for the potent hematopoietic recovery effects of low dose IL-12 when given before administration of myelosuppressive, or myeloablative, agents, especially radiation. Perhaps, like TPO derivatives, such as Peg-rHu-MGDF, IL-12 administration after myelosuppression works via inhibition of p53-dependent apoptosis [56]. Other possible mechanisms that can explain the ability of IL-12 to work after myelosuppression may be related to the stimulation of DNA repair mechanisms [57-59]. Studies are underway in our laboratory to elucidate the basis for the hematopoietic recovery effects when IL-12 is given after the administration of a myelosuppressive agent.

However, in contrast to the data reported herein, previous studies in both mice and humans reported IL-12 suppressed hematopoiesis and yielded considerable toxicity. How can these prior studies be reconciled with the present studies? First, in both mice and human studies repeated, and relatively high, dosing of IL-12 resulted in suppression of hematopoiesis and the induction of toxicity. These effects are related mainly to the production of interferon gamma (IFN-γ) [30,60-62]. Second, in murine studies where high and repeated dosing of IL-12 was used, IFN-γ was found to negatively regulate bone marrow hematopoiesis [61]. However, in the present studies, we have not used a high or repeated dose regimen. Instead we have utilized single, low dose regimens that promoted hematopoiesis without any apparent toxicity.

Moreover, because our regimens are intended to utilize IL-12 in conjunction with a myelosuppressive agent, such as radiation or chemotherapy, we do not expect to observe IL-12-induced myelosuppression or toxicity. As shown in the present studies, radiation or chemotherapy always results in a leukopenia, including lymphopenia. Importantly, in this scenario the mature blood cells that are responsible for the IL-12-induced production of IFN-γ, particularly the lymphocytes, are eradicated to a large extent by either radiation or chemotherapy. We suggest, therefore, that the leukopenia resulting from the myelosuppressive effects of radiation or chemotherapy interrupts the feedback loop between IL-12 and IFN-γ, thereby permitting IL-12 to promote hematopoeisis without the negative feedback imposed by IFN-γ. Moreover, since INF-γ appears to be the major cytokine responsible for IL-12-related suppression of hematopoiesis, as well as toxicity [30,60-62], the interruption of INF-γ production following the administration of myelosuppressive agents suggests that in future clinical trials of IL-12 as a hematological adjuvant cancer therapy, the hematopoietic-promoting activity of IL-12 should result in considerably less toxicity as compared to previous clinical studies. This notion has already been borne out by a recent study with AIDS patients with Kaposi's sarcoma undergoing HAART therapy. In this study, IL-12 was well tolerated when administered for up to 3 years to this myelosuppressed patient group [63].

If INF-γ-producing cells are fewer, or impaired, following myelosuppressive therapy, then what else might account for the antitumor effects found to accompany hematopoietic recovery in our studies? One notion is that when IL-12 is used in conjunction with a myelosuppressive therapy, the administration of exogenous IL-12 acts as a "danger signal" to induce the maturation of immature dendritic cells (DC) that survive myelosuppressive therapy. Furthermore, since immature DC are particularly suited to capture tumor-associated antigens, which are likely to be released from cancer cells following the cytotoxic action of a myelosuppressive agent, the resulting mature DC would be primed to produce effector T cells capable of mounting antitumor responses as hematopoietic recovery is also being achieved [64]. This notion is supported by the increased antitumor effects of IL-12 in later stage tumor model systems, as compared to early stage models (See Figures 4 and 8).

Given the remarkable preclinical results presented herein, our plans are to move IL-12 from the research level back into the clinic. We envision that IL-12 can be used clinically to prevent hematological toxicity, preserve bone marrow function and permit higher dose escalation of the cytotoxic agent, either radiation or chemotherapy. In the prophylactic use of IL-12, it may be important that this cytokine can be used before and/or near the time of radiation or chemotherapy without causing proliferation of the underlying cancer, as was shown consistently in these studies for the pre-post and post-only dosing of IL-12.

Moreover, IL-12 may be one of the best-suited cytokines for prophylactic use in the clinic. G-CSF and EPO are never used clinically before a primary therapy because of their ability to promote tumor growth [personal communication from D. Douer], and TPO and SCF, which are no longer being pursued clinically, also proliferate tumor cells [65,66]. It is noteworthy that a "black box" warning recently has been issued by the FDA for the use of erythropoiesis-stimulating agents (ESAs) [67], as these agents were found to decrease survival and promote tumor growth when used in cancer patients [68,69].

Finally, our studies presented here, which utilized lower doses and novel dosing schedules of IL-12 serve to underscore a theme that is becoming apparent as the pharmaceutical industry pursues biologics as drugs, especially pleiotropic cytokines. It appears that an important lesson can be learned from the past clinical failures of many biologic drugs. This lesson may be that "less is more," *i.e*., lower doses that effectively perform the required function are safer, and perhaps, even more efficacious. And what is also becoming apparent is that dose scheduling may be the key to successful biologic drug development, especially in the area of oncology.

## Conclusion

In summary, we have shown that precise scheduling and dosing of IL-12 in conjunction with radiation or chemotherapy can promote effective hematopoietic recovery in mice, while still providing a concomitant reduction in tumor volumes. Moreover, the studies presented reveal that low, non-repeated doses of IL-12 can be effective in generating these effects. Our results portend that despite its past clinical failure, IL-12 appears to have significant clinical potential as a hematological adjuvant cancer therapy.

## Competing interests

Lena A. Basile and Timothy K. Gallaher are principals of Neumedicines Inc. and declare that they have competing interests. The remaining authors, namely Darryl Shibata, Joseph D. Miller and Dan Douer, declare that they have no competing interests.

## Authors' contributions

LAB was involved in all aspects of the studies presented, LAB created the experimental design, directed the experiments, performed data analysis and prepared the manuscript, TKG performed analyses of the microarray data, JDM performed the statistical analyses, DS performed analysis of bone marrow specimens, and DD provided clinical relevance to the data. All authors have read and approved the final manuscript.

## Supplementary Material

Additional file 1 Statistical analysis of peripheral blood recovery following radiation or chemotherapy. Data file 1 provides the combined statistical data for peripheral blood recovery for both the EL4 and LL tumor models following radiation and the separate statistical data for peripheral blood recovery for the EL4 and LL tumor models following chemotherapy. The data file contains 5 tables, which list all the statistical results related to peripheral blood recovery (*i.e*., Tables 3, 4A-4B, 5 and 6). **Table 3: Statistical Data Corresponding to Blood Analysis for the Radiation Studies for the Combined Data Set from the EL4 and LL tumor models**. **Table 3. Radiation Experiment: Combined EL4 and LL Models**. This table lists the results of the multivariate (RMANOVA) and univariate (ANOVA) analyses performed on the individual blood cell dependent variables following the observation of Blood Cell Type*Group (p > .001) and Blood Cell Type*Group*Day (p < .001) interactions in the overall repeated measures RMANOVA utilizing all blood cell dependent variables, Days as the replicate and Group and Model as between groups independent variables. The presence of Group*Day interactions in the RMANOVAs shown here for each blood cell variable justified the individual ANOVAs on the days indicated, followed by Tukey tests on those days to determine which groups differed significantly, as indicated in the text. **Table 4A: Statistical Data Corresponding to Blood Analysis for Chemotherapy Studies of the EL4 Model**. **Table 4A. Chemotherapy Experiment: EL4 Lymphoma Model**. This table lists the results of the multivariate (MANOVA) and univariate (ANOVA) analyses performed on the individual blood cell dependent variables following the observation of Blood Cell Type*Group (p > .001) and Blood Cell Type*Group*Day (p < .001) interactions in the overall repeated measures RMANOVA utilizing all blood cell dependent variables, Days as the replicate and Group and Model as between groups independent variables. The presence of Group main effects in the MANOVAs shown here for each blood cell variable justified the individual ANOVAs on the days indicated, followed by Tukey tests on those days to determine which groups differed significantly, as indicated in the text. **Table 4B: Statistical Data Corresponding to Blood Analysis for Chemotherapy Studies of the LL Model**. **Table 4B. Chemotherapy Experiment: Lewis Lung Cancer Model**. This table lists the results of the multivariate (MANOVA) and univariate (ANOVA) analyses performed on the individual blood cell dependent variables following the observation of Blood Cell Type*Group (p > .001) and Blood Cell Type*Group*Day (p < .001) interactions in the overall repeated measures RMANOVA utilizing all blood cell dependent variables, Days as the replicate and Group and Model as between groups independent variables. The presence of Group main effects in the MANOVAs shown here for each blood cell variable justified the individual ANOVAs on the days indicated, followed by Tukey tests on those days to determine which groups differed significantly, as indicated in the text. **Table 5: Tukey Values for Radiation Studies**. **Table 5 **lists the statistically significant Tukey values for all blood groups in the radiation study which utilized combined blood data from the EL4 and LL tumor models. **Table 6: Tukey Values for Chemotherapy Studies**. **Table 6 **lists the statistically significant Tukey values for all blood groups in the chemotherapy study listed separately for the EL4 and LL tumor models. Click here for file

Additional file 2 Highly expressed genes related to secreted factors identified from the microarray analyses of the EL4 lymphoma and the Lewis lung cancer cell lines. Data file 2 contains a comparative listing of highly expressed genes from the Affymetrix microarray analyses of the EL4 lymphoma and Lewis Lung cancer tumor cell cells. The list only includes expressed genes related to secreted factors. There are two tables in this data file, namely Table 7A and 7B, which list the highly expressed genes found in the EL4 and LL cell lines, respectively. **Table 7A: Highly Expressed Genes Related to Secreted Factors Found in the EL4 Cell line via Affymetrix Gene Chip Analysis**. **Table 7A **lists the relative signal for each highly expressed gene that correspond to secreted factors, the probe set and the gene name for the EL4 lymphoma cell line. **Table 7b: Highly Expressed Genes Related to Secreted Factors Found in the Lewis Lung Cancer Cell line via Affymetrix Gene Chip Analysis**. **Table 7A **lists the relative signal for each highly expressed gene that correspond to secreted factors, the probe set and the gene name for the Lewis lung cancer cell line. Click here for file
