# Supplementary material for: Multilineage hematopoietic recovery with concomitant antitumor effects using low dose Interleukin-12 in myelosuppressed tumor-bearing mice
Source: J Transl Med. 2008 May 19;6:26. doi: 10.1186/1479-5876-6-26 (PMC2424034; doi:10.1186/1479-5876-6-26)
Supplement: Additional file 1 — Statistical analysis of peripheral blood recovery following radiation or chemotherapy. Data file 1 provides the combined statistical data for peripheral blood recovery for both the EL4 and LL tumor models following radiation and the separate statistical data for peripheral blood recovery for the EL4 and LL tumor models following chemotherapy. The data file contains 5 tables, which list all the statistical results related to peripheral blood recovery (i.e., Tables 3, 4A-4B, 5 and 6). Table 3: Statistical Data Corresponding to Blood Analysis for the Radiation Studies for the Combined Data Set from the EL4 and LL tumor models. Table 3. Radiation Experiment: Combined EL4 and LL Models. This table lists the results of the multivariate (RMANOVA) and univariate (ANOVA) analyses performed on the individual blood cell dependent variables following the observation of Blood Cell Type*Group (p > .001) and Blood Cell Type*Group*Day (p < .001) interactions in the overall repeated measures RMANOVA utilizing all blood cell dependent variables, Days as the replicate and Group and Model as between groups independent variables. The presence of Group*Day interactions in the RMANOVAs shown here for each blood cell variable justified the individual ANOVAs on the days indicated, followed by Tukey tests on those days to determine which groups differed significantly, as indicated in the text. Table 4A: Statistical Data Corresponding to Blood Analysis for Chemotherapy Studies of the EL4 Model. Table 4A. Chemotherapy Experiment: EL4 Lymphoma Model. This table lists the results of the multivariate (MANOVA) and univariate (ANOVA) analyses performed on the individual blood cell dependent variables following the observation of Blood Cell Type*Group (p > .001) and Blood Cell Type*Group*Day (p < .001) interactions in the overall repeated measures RMANOVA utilizing all blood cell dependent variables, Days as the replicate and Group and Model as between groups independent variables. The presence of G [file 1479-5876-6-26-S1.pdf]

Additional Data File 1

Table 3: Statistical Data Corresponding to Blood Analysis for the Radiation Studies for the Combined Data Set from the EL4 and LL tumor models

Blood cell	Analysis	Effect	Day
**NE**	RMANOVA	Gp.= p<.002	all
		Gp.*Day=p<.02	all
	ANOVA	Gp.= p<.001	7
		Gp.= p<.02	10
		Gp. = p<.03	14
		Gp. = p<.02	21
**LY**	RMANOVA	Gp.*Day=p<.03	all
	ANOVA	Gp.= p<.01	7
		Gp.= p<.005	10
		Gp.= p<.03	18
**MO**	RMANOVA	Gp.*Day=p<0.10	all (marginal)
	ANOVA	Gp = p<0.10	7 (marginal)
		Gp = p<0.02	14
**RBC**	RMANOVA	Gp. = p<.001	all
		Gp.*Day=p<.001	all
	ANOVA	Gp. = p<.03	4
		Gp. = p<.04	7
		Gp. = p<.04	14
		Gp. = p<.001	18
		Gp. = p<.001	21
**PLT**	RMANOVA	Gp. = p<.001	all
		Gp.*Day = p<.001	all
	ANOVA	Gp. = p< .001	14
		Gp. = p< .001	18
		Gp. = p< .001	21

Table 3. Radiation Experiment: Combined EL4 and LL Models. This table lists the results of the multivariate (RMANOVA) and univariate (ANOVA) analyses performed on the individual blood cell dependent variables following the observation of Blood Cell Type*Group (p>.001) and Blood Cell Type*Group*Day (p<.001) interactions in the overall repeated measures RMANOVA utilizing all blood cell dependent variables, Days as the replicate and Group and Model as between groups independent variables. The presence of Group*Day interactions in the RMANOVAs shown here for each blood cell variable justified the individual ANOVAs on the days indicated, followed by Tukey tests on those days to determine which groups differed significantly, as indicated in the text.

Table 4A: Statistical Data Corresponding to Blood Analysis for Chemotherapy Studies of the EL4 Model

Blood cell	Analysis	Effect	Day
**NE**	MANOVA	Gp.= p<.05	all
	ANOVA	Gp.= p<.07 marginal	7
**LY**	MANOVA	Gp.= p<.02	all
	ANOVA	Gp.= p<.001	4
	ANOVA	Gp.= p<.05	7
**MO**		n.s.	
**RBC**		n.s.	
**PLT**	MANOVA	Gp. = p<.01	all
	ANOVA	Gp. = p<.01	4
	ANOVA	Gp. = p<.01	7
	ANOVA	Gp. = p<.01	8

Table 4A. Chemotherapy Experiment: EL4 Lymphoma Model. This table lists the results of the multivariate (MANOVA) and univariate (ANOVA) analyses performed on the individual blood cell dependent variables following the observation of Blood Cell Type*Group (p>.001) and Blood Cell Type*Group*Day (p<.001) interactions in the overall repeated measures RMANOVA utilizing all blood cell dependent variables, Days as the replicate and Group and Model as between groups independent variables. The presence of Group main effects in the MANOVAs shown here for each blood cell variable justified the individual ANOVAs on the days indicated, followed by Tukey tests on those days to determine which groups differed significantly, as indicated in the text.

Table 4B: Statistical Data Corresponding to Blood Analysis for Chemotherapy Studies of the LL Model

Blood cell	Analysis	Effect	Day
**NE**	MANOVA	Gp.= p<.01	all
	ANOVA	Gp.= p<.01	4
	ANOVA	Gp.= p<.07 marginal	6
**LY**		n.s.	
**MO**		n.s.	
**RBC**	MANOVA	Gp.=p<.01	all
	ANOVA	Gp.=p<.01	4
	ANOVA	Gp.=p<.01	7
**PLT**	MANOVA	Gp.=p<.01	all
	ANOVA	Gp.=p<.01	6
		Gp.=p<.01	7

Table 4B. Chemotherapy Experiment: Lewis Lung Cancer Model. This table lists the results of the multivariate (MANOVA) and univariate (ANOVA) analyses performed on the individual blood cell dependent variables following the observation of Blood Cell Type*Group (p>.001) and Blood Cell Type*Group*Day (p<.001) interactions in the

overall repeated measures RMANOVA utilizing all blood cell dependent variables, Days as the replicate and Group and Model as between groups independent variables. The presence of Group main effects in the MANOVAs shown here for each blood cell variable justified the individual ANOVAs on the days indicated, followed by Tukey tests on those days to determine which groups differed significantly, as indicated in the text.

Table 5: Tukeys for Radiation Studies

Neutrophils

As compared to Vehicle:	Day	P-value
G-CSF	7	<0.001
IL-12 pre-post	14	<0.05
IL-12 pre-post	21	<0.05

Lymphocytes

As compared to Vehicle:	Day	P-value
IL-12 post-only	7	<0.05
IL-12 pre-post	18	<0.05

Monocytes

As compared to Vehicle:	Day	P-value
IL-12 pre-post	14	<0.05

Red Blood Cells

As compared to Vehicle:	Day	P-value
IL-12 pre-post	7	=0.05
IL-12 pre-post	14	<0.05
All cytokine groups	18	<0.001
G-CSF	21	<0.05
IL-12 pre-only	21	<0.005
IL-12 post-only	21	<0.05
IL-12 pre-post	21	<0.001

Platelets

As compared to Vehicle:	Day	P-value
IL-12 pre-post	14	<0.001
IL-12 post	14	<0.001
IL-12 pre-post	18	<0.001
IL-12 pre-only	18	<0.001
G-CSF	18	=0.001
IL-12 pre-post	21	<0.001
IL-12 pre-only	21	<0.01
IL-12 post-only	21	<0.005

As compared to G-CSF:	Day	P-value
IL-12 pre-post	14	=0.001
IL-12 pre-post	18	=0.001
IL-12 pre-only	18	<0.05
IL-12 pre-post	21	<0.001
IL-12 pre-only	21	=0.05
IL-12 post-only	21	<0.05

Table 6: Tukeys for Chemotherapy Studies

Neutrophils

Lewis Lung Cancer

As compared to Vehicle:	Day	P-value
G-CSF	2	<0.001
G-CSF	6	=0.05

Red Blood Cells

Lewis Lung Cancer

As compared to Vehicle:	Day	P-value
IL-12 post-only	4	=0.01
IL-12 pre-post	4	<0.05

Platelets

EL4 Lymphoma

As compared to Vehicle:	Day	P-value
IL-12 post-only	8	<0.05

As compared to G-CSF:	Day	P-value
IL-12 pre-post	4	<0.05
IL-12 post-only	7	<0.001
IL-12 post-only	8	<0.001
IL-12 pre-post	8	<0.001

Lewis Lung Cancer

As compared to Vehicle:	Day	P-value
G-CSF	7	=0.01
IL-12 pre-only	7	<0.001
IL-12 post-only	7	<0.001
IL-12 pre-post	7	<0.001

As compared to G-CSF:	Day	P-value
IL-12 pre-only	6	=0.01
IL-12 pre-post	6	=0.01
IL-12 pre-only	7	<0.01
IL-12 post-only	7	<0.001
IL-12 pre-post	7	<0.001
