# Supplementary material for: Multilineage hematopoietic recovery with concomitant antitumor effects using low dose Interleukin-12 in myelosuppressed tumor-bearing mice
Source: J Transl Med. 2008 May 19;6:26. doi: 10.1186/1479-5876-6-26 (PMC2424034; doi:10.1186/1479-5876-6-26)
Supplement: Additional file 2 — Highly expressed genes related to secreted factors identified from the microarray analyses of the EL4 lymphoma and the Lewis lung cancer cell lines. Data file 2 contains a comparative listing of highly expressed genes from the Affymetrix microarray analyses of the EL4 lymphoma and Lewis Lung cancer tumor cell cells. The list only includes expressed genes related to secreted factors. There are two tables in this data file, namely Table 7A and 7B, which list the highly expressed genes found in the EL4 and LL cell lines, respectively. Table 7A: Highly Expressed Genes Related to Secreted Factors Found in the EL4 Cell line via Affymetrix Gene Chip Analysis. Table 7A lists the relative signal for each highly expressed gene that correspond to secreted factors, the probe set and the gene name for the EL4 lymphoma cell line. Table 7b: Highly Expressed Genes Related to Secreted Factors Found in the Lewis Lung Cancer Cell line via Affymetrix Gene Chip Analysis. Table 7A lists the relative signal for each highly expressed gene that correspond to secreted factors, the probe set and the gene name for the Lewis lung cancer cell line. [file 1479-5876-6-26-S2.pdf]

Additional Data File 2

Table 7a

Probe	Signal	Gene	Gene Symbol
1434814_x_at	51256.2	glucose phosphate isomerase 1	Gpi1
1420997_a_at	49826.5	glucose phosphate isomerase 1	Gpi1
1450081_x_at	46432.2	glucose phosphate isomerase 1	Gpi1
1421375_a_at	12405.6	S100 calcium binding protein A6 (calcyclin)	S100a6
1415888_at	7515.1	hepatoma-derived growth factor	Hdgf
1448570_at	6735.7	glia maturation factor, beta	Gmfb
1419964_s_at	6065.9	hepatoma-derived growth factor	Hdgf
1420653_at	4919.2	transforming growth factor, beta 1	Tgfb1
1417069_a_at	2505.1	glia maturation factor, beta	Gmfb
1448571_a_at	2388.4	glia maturation factor, beta	Gmfb
1416696_at	2347.6	DNA segment, Chr 17, Wayne State University 104, expressed	D17Wsu104e
1420909_at	2159.5	vascular endothelial growth factor A	Vegfa
1426327_s_at	1946.5	ciliary neurotrophic factor /// zinc finger protein 91	Cntf /// Zfp91
1451803_a_at	1355.5	vascular endothelial growth factor B	Vegfb
1451959_a_at	1354.9	vascular endothelial growth factor A	Vegfa
1431686_a_at	1347.2	glia maturation factor, beta"	Gmfb
1419123_a_at	1244.7	platelet-derived growth factor, C polypeptide	Pdgfc
1449351_s_at	1215.3	platelet-derived growth factor, C polypeptide	Pdgfc
1448156_at	1132.6	trefoil factor 1	Tff1
1443856_at	1090.1	rabaptin, RAB GTPase binding effector protein 1	Rabep1
1451645_at	1031.2	fibrosin 1	Fbs1
1425154_a_at	541.7	colony stimulating factor 1 (macrophage)	Csf1

Table 7b

Probe	Signal	Gene	Gene Symbol
1449254_at	49389.3	secreted phosphoprotein 1	Spp1
1421375_at	41858.4	S100 calcium binding protein A6 (calcyclin)	S100a6
1434814_x_at	24161.7	glucose phosphate isomerase 1	Gpi1
1420997_at	21967.9	glucose phosphate isomerase 1	Gpi1
1450081_x_at	19166.1	glucose phosphate isomerase 1	Gpi1
1415855_at	13188.6	kit ligand	Kitl
1415888_at	9869.7	hepatoma-derived growth factor	Hdgf
1448570_at	8831	glia maturation factor, beta	Gmfb
1419964_s_at	8805	hepatoma-derived growth factor	Hdgf
1420909_at	5632.8	vascular endothelial growth factor A	Vegfa
1419209_at	5591.2	chemokine (C-X-C motif) ligand 1	Cxcl1
1460220_at	5148.3	colony stimulating factor 1 (macrophage)	Csf1
1448117_at	5040.6	kit ligand	Kitl
1451959_at	4188.3	vascular endothelial growth factor A	Vegfa
1419123_at	4046	platelet-derived growth factor, C polypeptide	Pdgfc
1449351_s_at	3873.1	platelet-derived growth factor, C polypeptide	Pdgfc
1421134_at	3832.3	amphiregulin	Areg
1416696_at	3613.6	DNA segment, Chr 17, Wayne State University 104, expressed	D17Wsu104e
1418350_at	3269.8	heparin-binding EGF-like growth factor	Hbegf
1417879_at	3252.5	neuron derived neurotrophic factor	Nenf
1448914_at	3099.8	colony stimulating factor 1 (macrophage)	Csf1
1422168_at	3080	brain derived neurotrophic factor	Bdnf
1417069_at	3027.6	glia maturation factor, beta	Gmfb
1426238_at	2636.1	bone morphogenetic protein 1	Bmp1
1448254_at	2602.9	pleiotrophin	Ptn
1448571_at	2522.8	glia maturation factor, beta	Gmfb
1422243_at	2254.3	fibroblast growth factor 7	Fgf7
1451803_at	2114.1	vascular endothelial growth factor B	Vegfb

Probe	Signal	Gene	Gene Symbol
1427457_at	1973.3	bone morphogenetic protein 1	Bmp1
1448439_at	1917.9	DNA segment, Chr 17, Wayne State University 104, expressed	D17Wsu104e
1425154_at	1841.7	colony stimulating factor 1 (macrophage)	Csf1
1431686_at	1841	glia maturation factor, beta	Gmfb
1426326_at	1750.9	zinc finger protein 91	Zfp91
1443856_at	1700.6	rabaptin, RAB GTPase binding effector protein 1	Rabep1
1448156_at	1624	trefoil factor 1	Tff1
1422169_at	1618.6	brain derived neurotrophic factor	Bdnf
1426649_at	1493.6	transmembrane protein with EGF-like and two follistatin-like domains	Tmeff1
1448440_x_at	1467.4	DNA segment, Chr 17, Wayne State University 104, expressed	D17Wsu104e
1416211_at	1463.9	pleiotrophin	Ptn
1425155_x_at	1375.8	colony stimulating factor 1 (macrophage)	Csf1
1419431_at	1338.2	epiregulin	Ereg
1418349_at	1266.1	heparin-binding EGF-like growth factor	Hbegf
1427448_at	1241.1	rabaptin, RAB GTPase binding effector protein 1	Rabep1
1421305_x_at	1148.2	rabaptin, RAB GTPase binding effector protein 1	Rabep1
1415854_at	1065.9	kit ligand	Kitl
1426327_s_at	1012.5	ciliary neurotrophic factor /// zinc finger protein 91	Cntf /// Zfp91
1420653_at	683.9	transforming growth factor, beta 1	Tgfb1
